# Natural Killer Cell Functions during the Innate Immune Response to Pathogenic Streptococci

**DOI:** 10.3389/fmicb.2017.01196

**Published:** 2017-06-29

**Authors:** Paul Lemire, Tristan Galbas, Jacques Thibodeau, Mariela Segura

**Affiliations:** ^1^Laboratory of Immunology of the Swine and Poultry Infectious Diseases Research Centre, Faculty of Veterinary Medicine, University of MontrealSt-Hyacinthe, QC, Canada; ^2^Laboratory of Molecular Immunology, Faculty of Medicine, University of MontrealMontreal, QC, Canada

**Keywords:** group B *Streptococcus*, *Streptococcus suis*, natural killer cells, dendritic cells, capsular polysaccharide

## Abstract

Dendritic cells (DCs) and NK cells play a crucial role in the first phase of host defense against infections. Group B *Streptococcus* (GBS) and *Streptococcus suis* are encapsulated streptococci causing severe systemic inflammation, leading to septicemia and meningitis. Yet, the involvement of NK cells in the innate immune response to encapsulated bacterial infection is poorly characterized. Here, it was observed that these two streptococcal species rapidly induce the release of IFN-γ and that NK cells are the major cell type responsible for this production during the acute phase of the infection. Albeit *S. suis* capacity to activate NK cells was lower than that of GBS, these cells partially contribute to *S. suis* systemic infection; mainly through amplification of the inflammatory loop. In contrast, such a role was not observed during GBS systemic infection. IFN-γ release by NK cells required the presence of DCs, which in turn had a synergistic effect on DC cytokine production. These responses were mainly mediated by direct DC-NK cell contact and partially dependent on soluble factors. Though IL-12 and LFA-1 were shown to be critical in *S. suis*-mediated activation of the DC-NK cell crosstalk, different or redundant molecular pathways modulate DC-NK interactions during GBS infection. The bacterial capsular polysaccharides also differently modulated NK cell activation. Together, these results demonstrated a role of NK cells in the innate immune response against encapsulated streptococcal infections; yet the molecular pathways governing NK activation seem to differ upon the pathogen and should not be generalized when studying bacterial infections.

## Introduction

*Streptococcus suis* and Group B *Streptococcus* (GBS, or *Streptococcus agalactiae*) are two encapsulated pathogens that induce similar pathologies, including septicemia and meningitis in animals and/or humans. Among ten GBS serotypes that have been characterized, type III GBS is the most common type in neonatal meningitis while type V GBS has long been recognized as a leading cause of invasive disease in adults (Le Doare and Heath, 2013). *S. suis* is not only a major swine pathogen but also emerging threat to human health, especially in Asian countries (Gottschalk et al., 2010; Fittipaldi et al., 2012). *S. suis* is now the leading cause of adult meningitis in Vietnam, the second in Thailand and the third in Hong Kong (Gottschalk et al., 2010). Among 35 *S. suis* serotypes that have been described, type 2 *S. suis* is the most virulent for both pigs and humans, and most of the studies have been performed with this serotype. In addition, type 14 *S. suis* is also emerging as a zoonotic agent (Goyette-Desjardins et al., 2014).

The capsular polysaccharide (CPS) defines the serotype and is considered a key virulence factor for both bacterial species (Cieslewicz et al., 2005; Maisey et al., 2008; Gottschalk et al., 2010; Fittipaldi et al., 2012). Indeed, these two streptococci are the sole Gram-positive bacteria harboring a side chain terminated by sialic acid in their CPS compositions. In spite of this and other CPS biochemical and structural similarities (Cieslewicz et al., 2005; Van Calsteren et al., 2010, 2013), GBS and *S. suis* pathogenic mechanisms and interplay with components of the immune system seem to radically differ (Segura et al., 1998; Maisey et al., 2008; Lecours et al., 2011; Fittipaldi et al., 2012; Lemire et al., 2012a,b; Segura, 2012). Experiments using non-encapsulated mutants have shown that type 2 and type 14 *S. suis* CPSs have a strong antiphagocytic effect and severely interfere with the release of cytokines by *S. suis*-infected antigen-presenting cells (APCs). In contrast, encapsulated type III or type V GBS are easily internalized by APCs and cytokine production is only partially modified or unaltered by the presence of CPS (Segura et al., 1998; Lecours et al., 2011; Lemire et al., 2012a,b, 2014; Roy et al., 2015).

Protective immunity requires the coordinated activation of both the innate and adaptive immune systems. Interactions between innate and adaptive immune effectors are essential for the efficient control of pathogens and often play an important role in ending immune responses which would otherwise eventually be harmful to the host. During infection, myeloid cells play a key role not only as first line of defense but also as key cells that contribute to the activation of other innate immune cells, such as natural killer (NK) cells. Dendritic cells (DCs) are recognized as the most powerful APCs that initiate immune responses against pathogens and are considered an essential link between innate and adaptive immunity. Consequently, the interactions between DCs and other immune cells can strongly influence the outcome of a disease, and more importantly the magnitude and phenotype of the ensuing adaptive immune response to the invading pathogen. NK cells are large granular lymphocytes recognized as playing a pivotal role in the innate immune response mainly against viruses. However, recent studies provided strong evidence for the participation of NK cells in the innate immune response to bacterial infections via the production of pro-inflammatory cytokines, such as interferon gamma (IFN-γ) and tumor necrosis factor-alpha (TNF-α) (Souza-Fonseca-Guimaraes et al., 2012; Adib-Conquy et al., 2014). Indeed, NK cells possess receptors allowing them to sense and respond to viral and bacterial patterns, including Toll-like receptors (TLRs) (Adib-Conquy et al., 2014).

Several studies have demonstrated that the crosstalk between DCs and NK cells can potentiate each other efficiency (Moretta et al., 2006). Stimulation of DCs drives the production of key NK cell-activating cytokines, such as interleukin (IL)-12 and IL-18, in addition to enhancing cell-cell contact between DCs and NK cells. This DC-NK cell crosstalk is known to be important in NK cell activation, IFN-γ production and acquisition of effector functions. In return, NK cells contribute to DC maturation and activation (Borg et al., 2004; Moretta et al., 2006). Nevertheless, few studies have addressed the role of DC-NK cell crosstalk, which might represent a major and early source of IFN-γ production, during streptococcal infections (Elhaik-Goldman et al., 2011; Bouwer et al., 2013; Lachance et al., 2013a). Indeed, among pro-inflammatory cytokines, IFN-γ has gained much interest over the years for its important beneficial role in controlling GBS infections. In fact, it was shown that IL-12 and IL-18 mediate their therapeutic effects by increasing IFN-γ production by responding immune cells (Cusumano et al., 1996, 2004; Mancuso et al., 1997). A protective role of IFN-γ was also shown in type III and type V GBS infections in mice. Furthermore, IFN-γ production is severely impaired during early life and might partly explain the susceptibility of neonates to GBS infection (Wilson, 1986; Cusumano et al., 1996). Similarly, clinical and epidemiological data from type 2 *S. suis*-infected patients showed that during the early phase of the disease, serum levels of IFN-γ, and other inflammatory cytokines, are extremely high (Ye et al., 2009). Mice experimentally infected with virulent type 2 *S. suis* strains showed increased systemic levels of IFN-γ expression early after infection (Lachance et al., 2013a). Albeit NK cells have been suggested as a potential source of IFN-γ production during either type III GBS or type 2 *S. suis* infections (Derrico and Goodrum, 1996; Lachance et al., 2013a), modulation of the DC-NK cell crosstalk by these two pathogenic streptococci has never been addressed before.

Based on these observations and previous findings on GBS and *S. suis* interactions with DCs, the hypothesis of this study is that GBS and *S. suis* drive NK cell production of IFN-γ and other inflammatory cytokines that depend on the formation of a DC-NK cell crosstalk. We also hypothesize that the bacterial CPSs differentially modulate these interactions. To this aim, we investigated *ex vivo* and *in vivo* the role of NK cells during the innate immune response against type III GBS or type 2 *S. suis. In vitro* DC-NK co-culture systems were used to further dissect the molecular pathways leading to NK cell activation and to evaluate the role of the CPS by studying different GBS or *S. suis* capsular serotypes and respective non-encapsulated mutants.

## Materials and methods

### Ethics statement

This study was carried out in accordance with the recommendations of the guidelines and policies of the Canadian Council on Animal Care (CCAC) and the principles set forth in the Guide for the Care and Use of Laboratory Animals, CCAC. The protocol was approved by the Animal Welfare Committee of the University of Montreal (protocol # Rech-1399).

### Bacterial strains and growth conditions

Bacterial strains used in this study are listed in Table 1. All strains were grown in Todd-Hewitt Broth (THB) or agar (THA) (Becton Dickinson, Mississauga, ON, Canada) or on sheep blood agar plates at 37°C for 18 h as previously described (Lemire et al., 2013; Calzas et al., 2015; Clarke et al., 2016). Briefly, isolated GBS or *S. suis* colonies were inoculated in 5 ml of THB and incubated for 8 h at 37°C with shaking. Working cultures were prepared by transferring 10 μl of 1/1,000 dilutions of 8 h-cultures into 30 ml of THB which was incubated overnight at 37°C with agitation. Early stationary phase bacteria were washed twice with PBS pH 7.3 before being appropriately diluted in fresh medium to desired inoculum concentrations. The number of CFU/ml in the final suspensions was determined by plating serial dilutions of working cultures on THA using an Autoplate 4000 Automated Spiral Plater (Spiral Biotech, Norwood, MA).

**Table 1:** Bacterial strains used in this study.

**Strains**	**General characteristics**	**Source/reference**
**Group B** ***Streptococcus***		
COH-1	Wild-type, encapsulated, and virulent strain isolated from an infant with septicemia and meningitis in United States of America. Serotype III.	Tettelin et al., 2005
Δ*cpsE*	Non-encapsulated mutant derived from strain COH-1. Deletion of the *cpsE* gene.	Lemire et al., 2012a
CJB111	Wild-type, encapsulated, and virulent strain isolated from a neonate with septicemia in United States of America. Serotype V.	Tettelin et al., 2005
Δ*cpsE*	Non-encapsulated mutant derived from strain CJB111. Deletion of the *cpsE* gene.	Lemire et al., 2014
***Streptococcus suis***		
P1/7	Wild-type, encapsulated, and virulent strain isolated from a pig with meningitis in United Kingdom. Serotype 2.	Slater et al., 2003
Δ*cpsF*	Non-encapsulated mutant derived from strain P1/7. Deletion of the *cpsF* gene.	Lecours et al., 2011
DAN13730	Wild-type, encapsulated, and virulent strain isolated from a human with meningitis in Netherlands. Serotype 14.	Gottschalk et al., 1989
Δ*cpsB*	Non-encapsulated mutant derived from strain DAN13730. Deletion of the *cpsB* gene.	Roy et al., 2015

### Generation of bone marrow-derived dendritic cells

Bone marrow-derived DCs were generated from C57BL/6 female mice 5-week-old (Charles River Laboratories, St-Constant, QC, Canada) according to a technique described elsewhere (Lecours et al., 2011; Lemire et al., 2012a,b; Clarke et al., 2016). Briefly, after red blood cell lysis, total bone marrow cells (2.5 × 10^5^ cells/ml) were cultured in complete medium consisting of RPMI 1640 supplemented with 5% heat-inactivated FBS, 10 mM HEPES, 20 μg/ml gentamycin, 100 U/ml penicillin-streptomycin, 2 mM L-glutamine, and 50 μM 2-β-mercaptoethanol. All reagents were from Life Technologies (Burlington, ON, Canada). Complete medium was complemented with 20% granulocyte-macrophage colony-stimulating factor (GM-CSF) from a mouse GM-CSF transfected cell line (Ag8653) as a source of GM-CSF. Cells were cultured for 7 days at 37°C with 5% CO_2_. On day 7, clusters were harvested and subcultured overnight to remove adherent cells. Non-adherent cells were collected on day 8 and used as immature DCs for the studies. Cell purity was routinely ≥ 88% CD11c^+high^ cells as determined by FACS analysis and as previously reported (Lecours et al., 2011; Lemire et al., 2012a,b).

### Culture of splenic NK cells

Untouched NK cells were purified from naive C57BL/6 mice by negative selection using NK cell isolation kit II microbeads and magnetically-activated cell sorting (MACS; Miltenyi Biotec, Auburn, CA). Purified NK cells were either used as freshly isolated cells or expanded *in vitro* for 8 days at 37°C with 5% CO_2_ in complete RPMI 1640 medium supplemented with 500 ng/ml of recombinant mouse IL-2 (Miltenyi Biotec) as previously reported (Sanabria et al., 2008; Pontiroli et al., 2012; Lachance et al., 2013a; Yea et al., 2014). The resulting NK cell purity of expanded cultures was routinely > 93% NK1.1^+^ and CD3^-^ as determined by FACS analysis (Figure S1).

### DC and NK cell co-cultures

DC and NK cell (DC-NK) co-cultures were established at a ratio of 1:3 in complete, antibiotic-free medium. This ratio was determined after standardization with different ratios of DCs and NK cells (Figure S2). The ratio of 1:3 was chosen as allowed to obtain a significant production of IFN-γ using a lower number of NK cells and thus reducing the number of animals required for the experiments. Co-cultures were infected with 2.5 × 10^5^ CFU of different GBS or *S. suis* strains (initial multiplicity of infection [MOI]:1 to DCs). As previously standardized (Lemire et al., 2012a,b, 2013, 2014; Lachance et al., 2013a; Clarke et al., 2016; Lecours et al., 2016), after bacterium-cell contact, antibiotics were added to the cultures to prevent cell toxicity and obtain an optimal antigenic stimulus, and thus DC activation for each pathogen. Supernatants were collected after a final 14 h of incubation. Non-stimulated cells (medium alone) served as negative control. In addition, single DC or NK cell cultures were also included as controls. In selected experiments, a Transwell® system was incorporated to the DC-NK co-cultures. A Transwell® insert with a 0.4 μM pore size polycarbonate membrane was placed in 24-well flat bottom Nunc plates (Fisher Scientific, Ottawa, ON, Canada). NK cells were placed in the lower compartment. DCs were placed in the upper compartment of the Transwell® insert. GBS or *S. suis* strains (initial MOI:1 to DCs) were added to both, the upper and the lower compartments, and co-cultures were incubated as described above. In some experiments, NK cells were cultured alone in the presence of non-diluted supernatants obtained from 14 h-infected DCs at an initial MOI:1.

For inhibition assays, DCs and/or NK cells were pre-treated 1 h prior to bacterial infection with anti-mouse IL-12 (20 μg/ml; clone C17.8, rat IgG_2a_) anti-mouse CD314/NKG2D (10 μg/ml; clone C7, Armenian Hamster IgG), anti-mouse CD11a/LFA-1 (10 μg/ml; clone M17/4, rat IgG_2a_) or isotype controls (rat IgG_2a_ and Armenian hamster IgG). All antibodies were from eBioscience (San Diego, CA). Doses of neutralizing antibodies were selected in pre-tests and/or based on the literature (Guan et al., 2007; Humann and Lenz, 2010; Jiao et al., 2011; Bouwer et al., 2013).

All solutions and bacterial preparations used in these experiments were tested for the absence of endotoxin contamination using a *Limulus* amebocyte lysate test (Pyrotell, STV, Cape Cod, MA) with a sensitivity limit of 0.03 EU/ml.

### *Ex vivo* analysis of total splenocytes

C57BL/6 female, 5-week-old mice (Charles River Laboratories) were injected intraperitoneally (i.p.) with a dose of 1 × 10^7^ CFU of type III GBS strain COH-1 or 5 × 10^7^ CFU of type 2 *S. suis* strain P1/7. Mouse models of infection, including injection dose, for these two bacterial strains have been previously standardized (Dominguez-Punaro Mde et al., 2008; Lachance et al., 2013a,b; Lemire et al., 2013; Clarke et al., 2016; Lecours et al., 2016). Control mice were injected with the vehicle solution (sterile THB). Spleens from infected mice with clinical signs of disease and positive bacteremia (1–5 × 10^8^ CFU/ml of blood) were harvested 6 h post-infection (*n* = 3 per group × 3 individual experimental infections), perfused with complete RPMI medium (without antibiotics), teased apart, and pressed gently through a sterile fine wire mesh. After red blood cells lysis and washing, total splenocytes were counted and plated at a concentration of 5 × 10^6^ cells/ml in complete RPMI medium in 24-well flat bottom plates, and incubated at 37°C with 5% CO_2_ for 5 and 14 h. Antibiotics were added to the cultures to prevent cell toxicity as described (Lachance et al., 2013a; Lemire et al., 2013; Clarke et al., 2016; Lecours et al., 2016). Total splenocytes from control (placebo) animals were similarly treated. Brefeldin A (3 μg/ml) was added during the last 5 h, and total splenocytes were analyzed by intracellular FACS (see below). In selected experiments, Brefeldin A (400 μg/ml) was injected i.p. directly to mice 5 h prior to spleen collection and cells were directly analyzed by intracellular FACS (without an *ex vivo* incubation step).

### *In vivo* NK1.1^+^ cell depletion

To deplete NK1.1^+^ cells, supernatant of anti-NK1.1 mouse monoclonal antibody (mAb) was produced by the hybridoma cell line PK-136 (ATCC HB-1991TM). NK1.1^+^ cell depletion started 4 d prior to bacterial infection by i.p. injections of 1 ml of supernatant (1 mg mAb) or isotype control in culture medium (mock-treated). Injections were repeated 2 and 1 days before bacterial infection. NK1.1^+^ cell numbers post-depletion were evaluated by FACS (as described Frenkel et al., 2016; Benigni et al., 2017) in the spleens of mock-treated and mAb treated-animals. NK1.1^+^ cells were depleted beyond >75% (Figure S3). At the day of the infection, mice were injected i.p. with different doses of type III GBS strain COH-1 or type 2 *S. suis* strain P1/7. Mice were closely monitored to record mortality and clinical signs of disease, such as depression, rough appearance of hair coat, and swollen eyes. Mice exhibiting extreme lethargy were considered moribund and were humanely euthanized. To determine the level of infection, numbers of viable bacteria in blood were quantified at 6, 12, and 24 h post-infection. Blood samples were collected from the tail, serially diluted in PBS and plated onto THA plates as described above. Colonies were counted and expressed as CFU/ml of blood. To measure plasma cytokine levels, mice were euthanized at 24 h post-infection and blood collected by cardiac puncture. Non-infected mice were used as negative controls. Plasma was conserved at −80°C for cytokine analyses. Numbers of animals per group are displayed in figure legends.

### Cytokine quantification by ELISA

Levels of IL-6, IL-10, IL-12p40, IL-12p70, IFN-γ, TNF-α, C-C motif chemokine ligand (CCL) 2, CCL5, C-X-C motif chemokine ligand (CXCL) 9, CXCL10, and granulocyte-colony stimulating factor (G-CSF) in cell culture supernatants were measured by sandwich ELISA using pair-matched antibodies from R&D Systems (Minneapolis, MN) or eBioscience, according to the manufacturer's recommendations. Two-fold dilutions of recombinant mouse cytokines were used to generate standard curves. Sample dilutions giving OD readings in the linear portion of the appropriate standard curve were used to quantify the levels of each cytokine. The results are from at least three independent ELISA measurements.

### FACS analysis

For cell surface staining of total spleen cells, 10^6^ cells were washed and treated for 15 min on ice with FcR-blocking reagent (FcγIII/II Rc Ab) in sorting buffer (PBS-1% FBS). Blocked cells were then incubated with different combinations of the following antibodies: PECy7-conjugated anti-NK1.1 mAb (clone PK-136), PE-conjugated anti-CD19 (clone 6D5), FITC-conjugated anti-CD3 (clone 17A2), and/or PE-conjugated anti-CD69 mAb (clone H1.2F3) for 30 min on ice. For intracellular staining, cells were fixed, permeabilized, and incubated with APC-conjugated anti-mouse IFN-γ (clone XMG1.2) or APC-conjugated anti-mouse TNF-α (clone MP6-259 XT22) for 45 min at room temperature. All reagents were from BD Biosciences (Mississauga, ON, Canada). FACS was either performed using an Accuri™ C6 instrument or a FACSCanto II instrument (BD Biosciences). For acquisition, 30,000–70,000 events were acquired per sample and data analyses were performed using BD Accuri C6 software or FACSDiva™ software. Quadrants were drawn based on control stains and were plotted on logarithmic scales. Fluorescence Minus One (FMO) control staining was performed for proper analysis and gating of target cells.

### Statistical analysis

Data are expressed as mean ± SEM and analyzed for significance using Student's unpaired *t*-test. The Kaplan-Meier method and log-rank Mantel-Cox tests were used to compare mouse survival rates of the studied groups. All analyses were performed using the Sigma Plot System (v.11.0; Systat Software). A *P* < 0.05 was considered as statistically significant.

## Results

### NK cells rapidly produce IFN-γ in response to either GBS or *S. suis*, yet inter-species differences are observed

**Figure 1:**
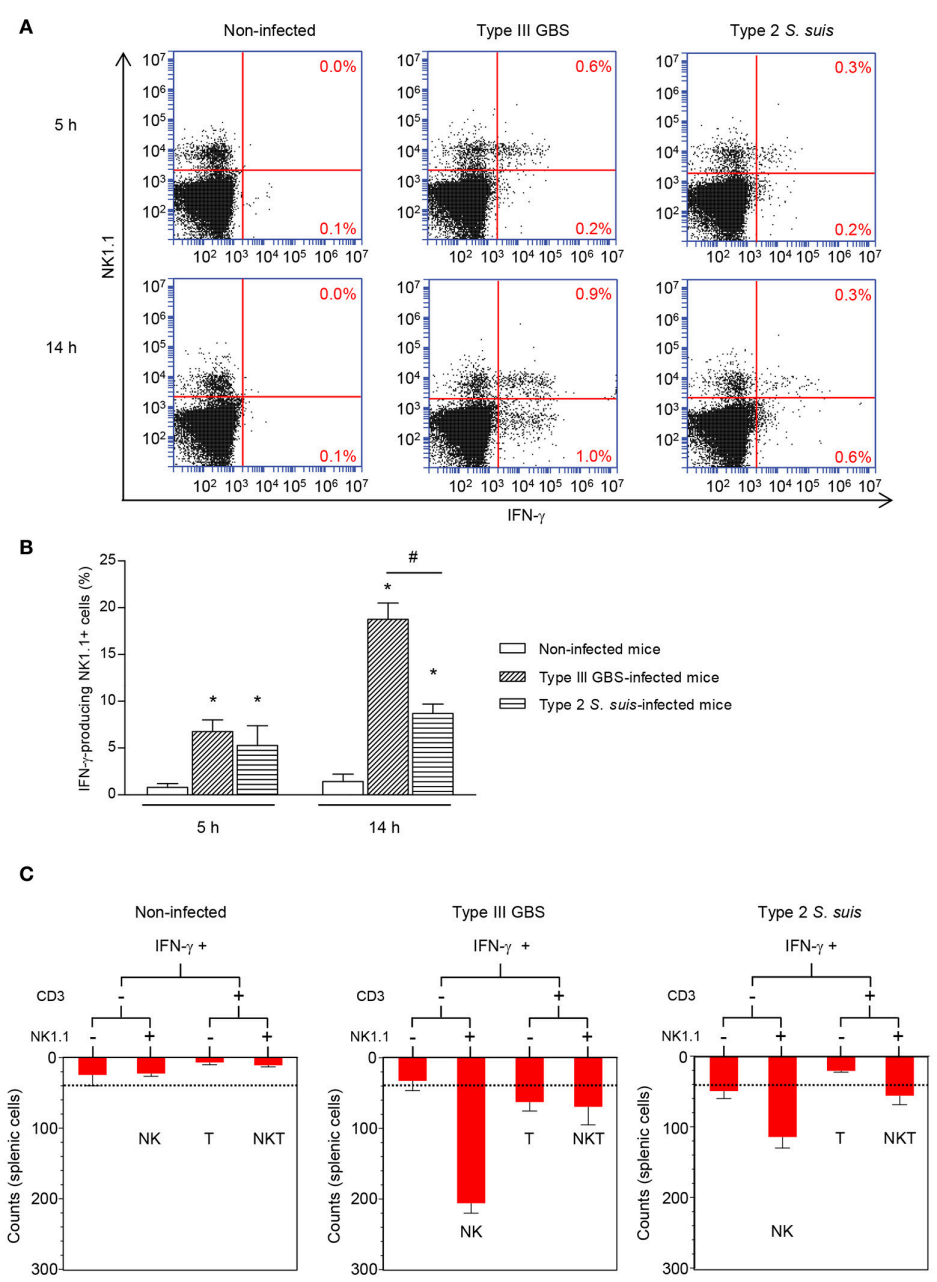
*Ex vivo* analyses of cellular sources of IFN-γ during the acute phase of streptococcal infections. Mice were infected intraperitoneally with a dose of 1 × 10^7^ of type III GBS wild-type strain COH-1 or 5 × 10^7^ CFU of type 2 *S. suis* wild-type strain P1/7 (*n* = 3 per group × 3 individual experimental infections). Spleens were harvested 6 h post-infection and total splenocytes plated at 5 × 10^6^ cells/well. Non-stimulated cells from mock-infected animals served as negative control for basal expression (Non-infected). Total splenocytes were incubated for 5 or 14 h with brefeldin A (3 μg/ml) added during the last 5 h of incubation. Cells were harvested and intracellularly stained for IFN-γ in combination with NK1.1 surface staining **(A,B)** or in combination with several surface markers for multi-parametric FACS analysis **(C)**. For **(B,C)**, to improve detection, IFN-γ expression was analyzed on 10,000 NK1.1^+^ **(B)** or 5,000 NK1.1^+^ **(C)** gated cells and data expressed as means ± SEM from three different experimental infections. ^*^P < 0.05, indicates statistically significant difference compared to non-infected control mice. ^#^*P* < 0.05, indicates statistically significant difference between type III GBS- and type 2 *S. suis*-infected mice. For **(C)**, results show IFN-γ^+^ cells within different spleen cell populations at 5 h. Dot-line indicates an increase in IFN-γ production over non-infected controls.

Before characterizing the mechanisms underlying NK cell activation in response to GBS or *S. suis*, we were interested in understanding the importance of NK cells in the acute phase of systemic infections by these two pathogens. To this aim, spleens from infected mice were collected 6 h of post-infection, at the onset of the first clinical signs of systemic disease and under comparable bacteremia levels for both pathogens (Figure S4). As plasma levels of IFN-γ are in general very low and barely detectable by ELISA (Teti et al., 1993; Cusumano et al., 1996; Mancuso et al., 1997; Dominguez-Punaro Mde et al., 2008; Lemire et al., 2013), intracellular levels of this cytokine were analyzed *ex vivo* in total splenocyte cultures, which also allows identification of cellular sources. As shown in Figures 1A,B, following infection with type III GBS, the number of IFN-γ-producing NK1.1^+^ cells increased overtime in infected mice and was significantly higher than that of the non-infected controls. Indeed, the NK1.1^+^ cells were shown to be the major producers of IFN-γ after 5 h of *ex vivo* incubation; however, at later time points (14 h of *ex vivo* incubation) the % of INF-γ-secreting NK 1.1^–^ cells also increased, suggesting a role of NK cells in the early boost of IFN-γ which is then amplified by other immune cells during type III GBS infection (Figure 1A).

As NK1.1^+^ cells include NK and NKT cells, to specifically dissect the role of NK cells and the potential contribution of other immune cells to IFN-γ production, multi-parametric intracellular FACS analyses of total splenocytes from type III GBS-infected mice were performed. NK cells (NK1.1^+^CD3^–^) represented ~75% of the IFN-γ production after 5 h of *ex vivo* incubation (Figure 1C) whereas NKT (NK1.1^+^CD3^+^) and T cells (CD3^+^) accounted each for less than 15 and 7.5% of this production, respectively. B cells (CD19^+^ cells) did not produce significant levels of this cytokine (data not shown).

The pattern of IFN-γ production after type 2 *S. suis*-infection differed from than observed for type III GBS (Figure 1A). First, the total number of IFN-γ-producing splenocytes was lower than that observed for type III GBS. Albeit NK1.1^+^ cells were also important producers of this cytokine, levels were low and did not seem to increase over time (Figure 1A). Indeed, by analyzing 10,000 NK1.1^+^ gated cells, a significant increase in IFN-γ production was observed compared to uninfected controls. However, and in contrast to type III GBS, this production remained stable across time (Figure 1B). In type 2 *S. suis*-infected mice, NK cells (NK1.1^+^CD3^–^) represented ~66% of the IFN-γ production after 5 h of *ex vivo* incubation. NKT cells (NK1.1^+^CD3^+^) were the second most important IFN-γ-producing cells (33%), while T and B cells produced no significant levels of IFN-γ at early time points (Figure 1C and data not shown).

Overall, these data suggest that NK cells produce an early IFN-γ response after either GBS or *S. suis* infection, yet inter-species differences are observed.

### Streptococcal infections increase surface expression of CD69 but fail to induce TNF-α production by NK1.1^+^ cells

**Figure 2:**
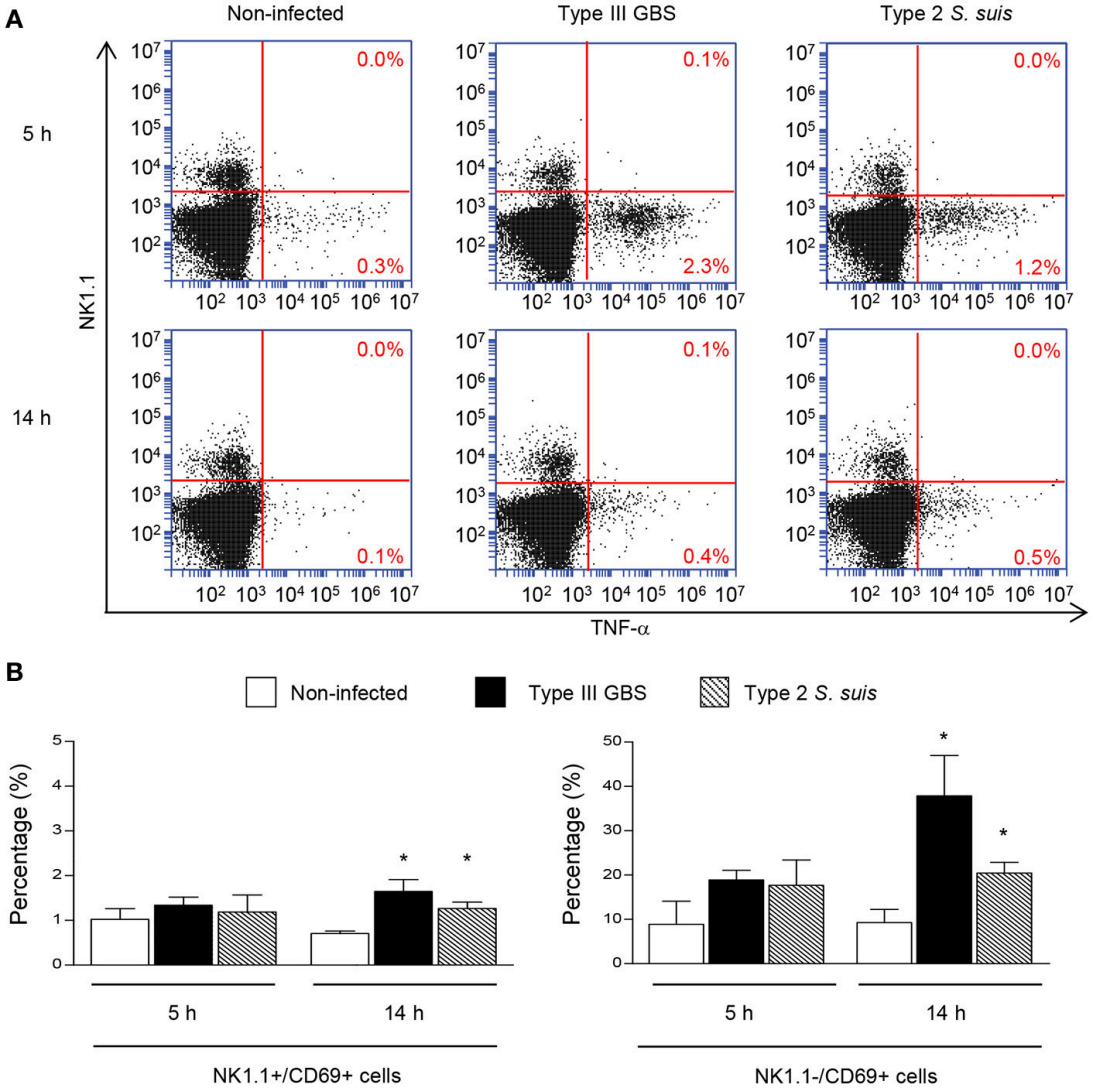
TNF-α production and CD69 expression by NK1.1^+^ cells during the acute phase of streptococcal infections. Mice were infected intraperitoneally with a dose of 1 × 10^7^ of type III GBS wild-type strain COH-1 or 5 × 10^7^ CFU of type 2 *S. suis* wild-type strain P1/7 (*n* = 3 per group × 3 individual experimental infections). Spleens were harvested 6 h post-infection and total splenocytes plated at 5 × 10^6^ cells/well. Non-stimulated cells from mock-infected animals served as negative control for basal expression (Non-infected). Total splenocytes were incubated for 5 or 14 h with brefeldin A (3 μg/ml) added during the last 5 h of incubation. Cells were harvested and intracellularly stained for TNF-α **(A)** or surface stained for CD69 **(B)** in combination with NK1.1 surface staining. **(A)** % of TNF-α^+^ cells within the NK1.1^−^ and the NK1.1^+^ cell populations are indicated. Representative data from three different experimental infections. **(B)** % of CD69^+^ cells within the NK1.1^+^ or the NK1.1^–^ cell populations of the spleen. Data are expressed as means ± SEM from three different experimental infections. ^*^P < 0.05, indicates statistically significant difference compared to non-infected control mice.

To measure the ability of GBS or *S. suis* to induce optimal activation of NK1.1^+^ cells, we measure surface expression of CD69 and the production of TNF-α, a cytokine described to be produced by these cells in the context of infection (Souza-Fonseca-Guimaraes et al., 2012). An early increase in TNF-α production was observed in total splenocytes from either type III GBS- or type 2 *S. suis*-infected mice. This production decreased over time (Figure 2A). Nevertheless, NK1.1^+^ cells were negative for TNF-α intracellular staining, suggesting that this cytokine production was generated mainly by cells of the innate immune system within the NK1.1^–^ population during a streptococcal infection. It should be mentioned that neither the analysis of 10,000 NK1.1^+^ gated cells nor the analysis of NK1.1^+^ cells directly *in vivo* (without an *ex vivo* incubation step) allowed detection of a significant production of TNF-α by NK1.1^+^ cells (data not shown). Total splenocytes from infected animals also showed a time-dependent increase in surface expression of the early activation marker CD69 (Figure 2B). CD69 expression was observed in both NK1.1^+^ and NK1.1^−^ spleen sub-populations (Figure 2B).

**Figure 3:**
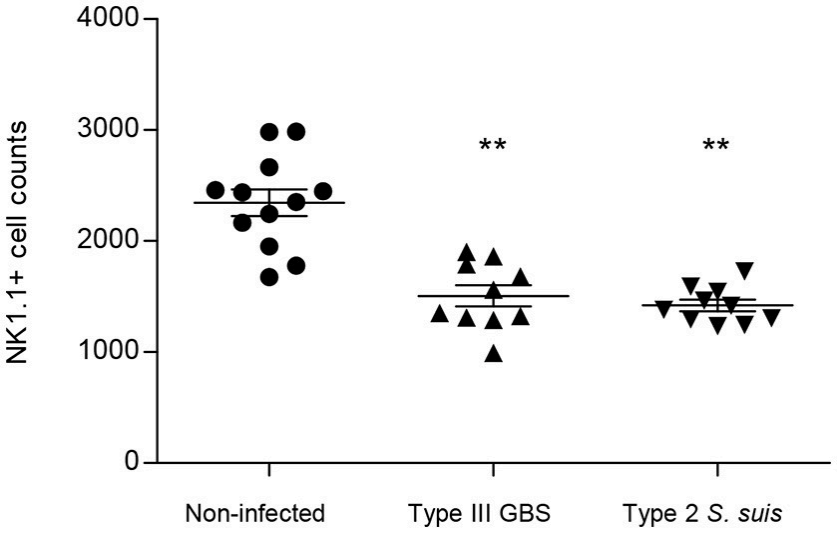
NK1.1^+^ population decreases after GBS or *S. suis* infection. Mice were infected intraperitoneally with a dose of 1 × 10^7^ of type III GBS wild-type strain COH-1 or 5 × 10^7^ CFU of type 2 *S. suis* wild-type strain P1/7 (*n* = 10 per group). Non-infected (placebo) animals served as controls (*n* = 12). Spleens were harvested 6 h post-infection and stained for NK1.1^+^ cell population. For analysis, 70,000 total events were acquired per sample and data are expressed as individual mouse values. ^**^P < 0.01, indicates statistically significant difference compared to non-infected control mice.

Taken as a whole, *ex vivo* analyses of total splenocytes from infected mice suggest that NK cells rapidly produce IFN-γ, increase CD69 expression at their surface but do not contribute to the total TNF-α production by infected splenic cells. Yet, type 2 *S. suis*-infected mice showed an overall lower innate immune response compared to a similar systemic infection with type III GBS. Interestingly, a reduction in the percentage of NK1.1^+^ cells was noticed following infection by GBS or *S. suis*. By analyzing the quantity of NK1.1^+^ cells after 6 h of *in vivo* infection, a significant decrease in the quantity of NK1.1^+^ cells (around 30–40%, *P* < 0.01) compared to non-infected controls was observed for both pathogens (Figure 3). These results suggest either a trafficking of NK1.1^+^ cells to the infection sites or could be a consequence of their apoptosis (Venet et al., 2010).

### Depletion of NK1.1^+^ cells promotes survival of mice during *S. suis* infection but not during GBS infection

**Figure 4:**
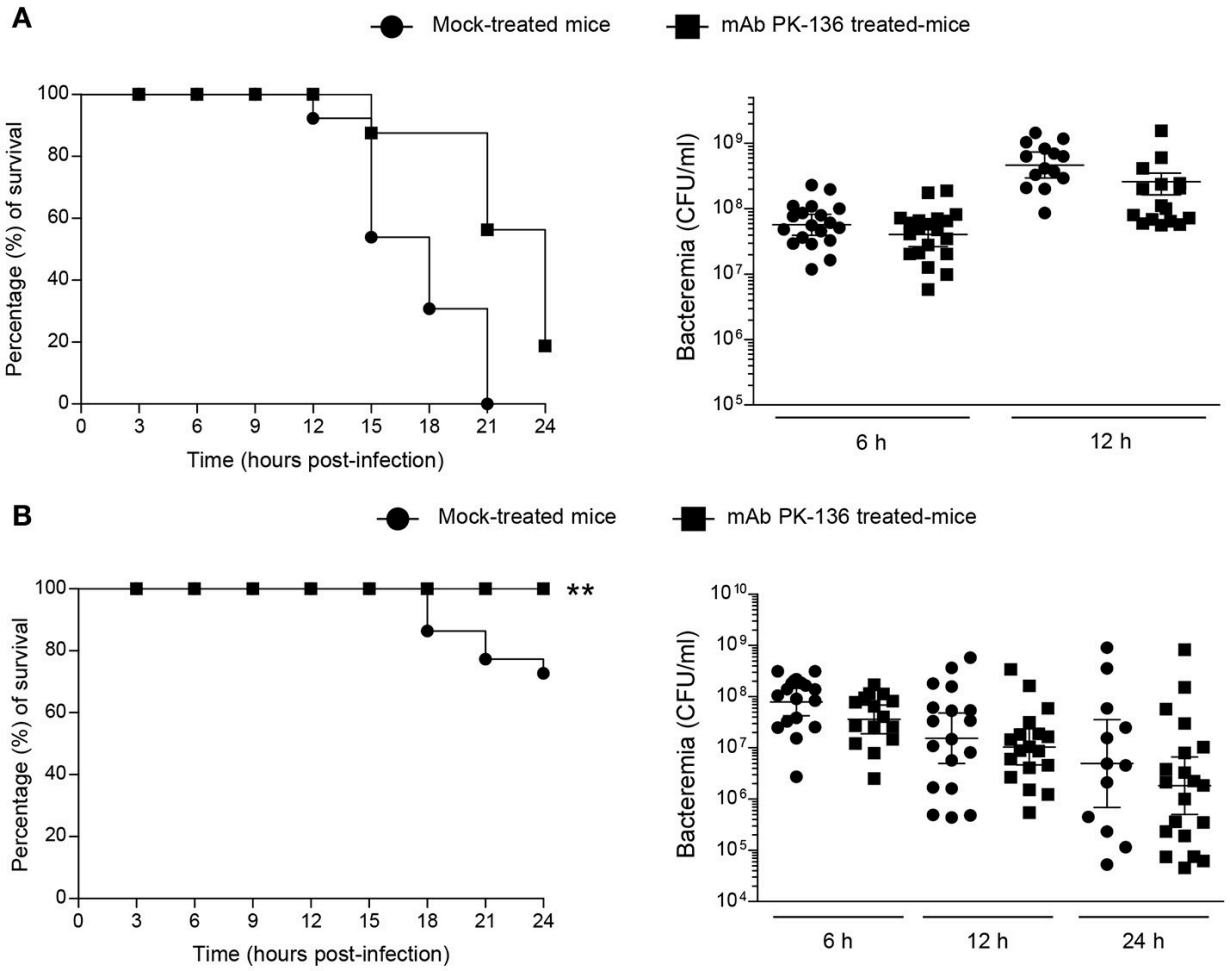
Effect of *in vivo* NK1.1^+^ cell depletion on GBS or *S. suis* infection is dose-independent or –dependent, respectively. Mice were either mock-treated or pre-treated with PK-136 monoclonal antibody (mAb) supernatant by 3 injections at days −4, −2, and −1 prior to infection. Twenty-four hours after the last injection (day 0), mice were intraperitoneally infected with a dose of 1 × 10^7^ CFU of type III GBS wild-type strain COH-1 or 5 × 10^7^ CFU of type 2 *S. suis* wild-type strain P1/7. Survival and levels of bacteremia of GBS-infected mice **(A)** or *S. suis-*infected mice **(B)** were monitored during 24 h (*n* = 20, results combined from two experiments). Blood samples were collected from the tail at 6, 12, and 24 h post-infection, and plated onto THB agar plates. Colonies were counted and data expressed as CFU/ml of blood. ^**^P < 0.01, indicates statistically significant differences between mock-treated and mAb PK136-treated mice infected with *S. suis* **(B)** according to the log-rank test (Mantel-Cox). For different doses of infection, refer to Supplementary Figure S5.

To determine if NK cells play a beneficial or detrimental role in the pathogenesis of streptococcal infections, we depleted NK1.1^+^ cells by anti-NK1.1 (clone PK-136) mAb-treatment prior to infection with GBS or *S. suis*. In spite of carefully standardized mouse models of infection (Figure S4), bacterial growth rates in blood might diverge between the two streptococcal species over 12 h of infection and the outcome of the treatment could be affected by bacteremia levels. As such, three different infectious doses were evaluated in this set of experiments (Figure 4 and Figure S5**)**.

As shown in Figure 4A, NK1.1^+^ cell depletion slightly delayed mouse death after systemic infection but does not influence the overall outcome of GBS infection at a dose of 1 × 10^7^ CFU, and almost all the mice died at 24 h post-infection. Moreover, the absence of NK1.1^+^ cells had no effect on bacteremia levels after 6 or 12 h post-infection (Figure 4A). Previous experiments demonstrated that a lower dose (1 × 10^6^ CFU) is not sufficient to cause clinical signs (Figure S4) and mice rapidly clear GBS (Lemire et al., 2013; Clarke et al., 2016). The depletion of NK1.1^+^ cells had no impact in the clearance of the infection at this dose or at an intermediate dose (3 × 10^6^ CFU) either (Figure S5A).

On the other hand, depletion of NK1.1^+^ cells partially reduced mortality in *S. suis*-infected mice, at least during the first 24 h of infection with a dose of 5 × 10^7^ CFU (Figure 4B). Due to the possibility of renewal in the pool of NK1.1^+^ cells after 48 h of the last dose of mAb treatment, we only focussed on the first 24 h of *S. suis* infection. Indeed, 30% of mock-treated mice died after 24 h post-infection compared to 0% in the NK1.1^+^ cell-depleted group (*P* < 0.005). Like GBS, the levels of bacteremia after 6, 12, or 24 h post-infection were similar between the two groups (Figure 4B). No significant differences were observed at lower *S. suis* infectious doses (<5 × 10^7^ CFU, Figure S5B), and no or very low mortality was recorded for both groups at 24 h post-infection. Higher doses (>5 × 10^7^) were lethal early after infection.

### NK1.1^+^ cells participate in the amplification of the inflammatory loop during *S. suis* infection

**Figure 5:**
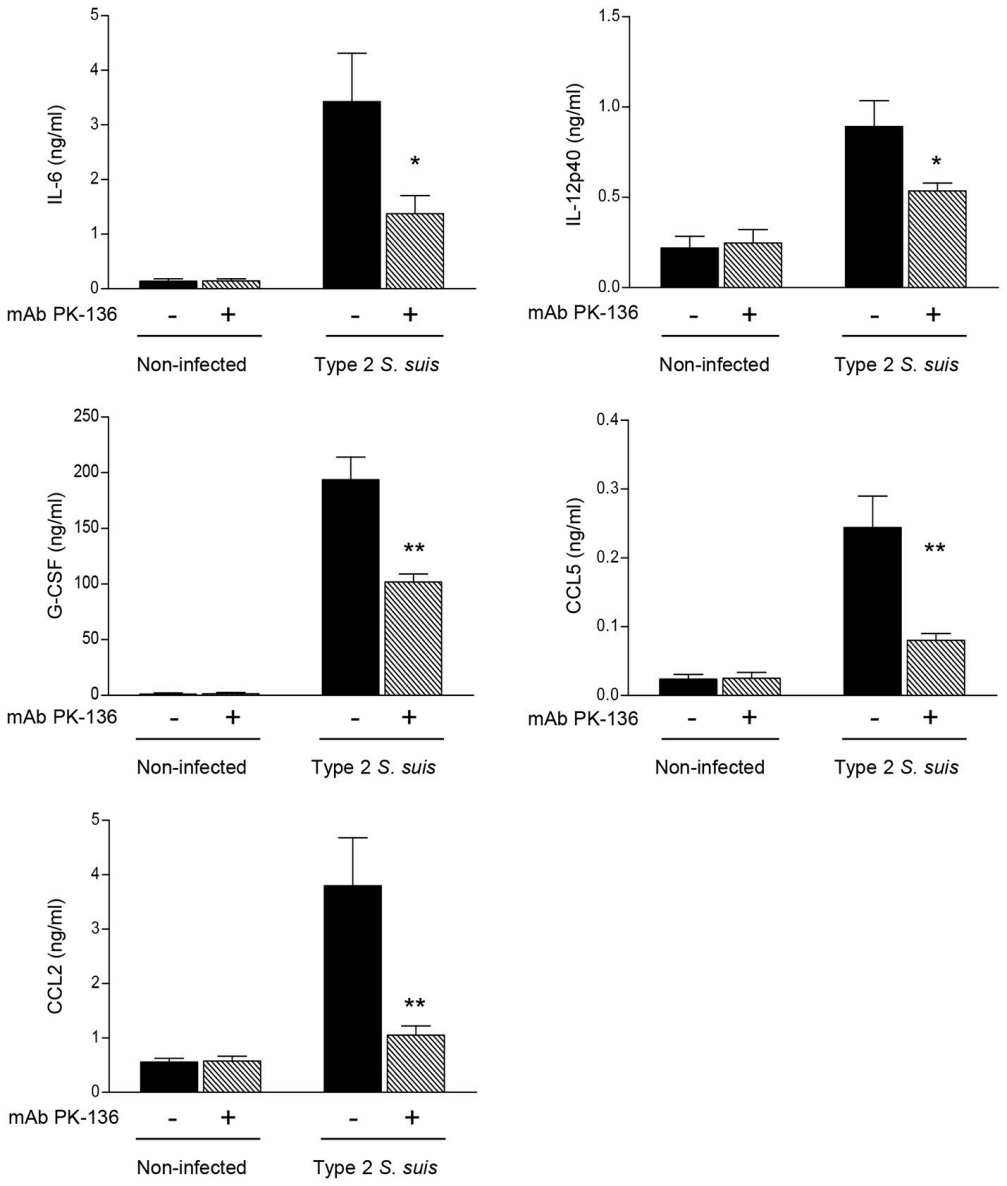
*In vivo* production of pro-inflammatory cytokines and chemokines is reduced in NK1.1^+^ cell depleted-mice infected with *S. suis*. Mice were either mock-treated or pre-treated with PK-136 monoclonal antibody (mAb) supernatant by 3 injections at days -4, -2, and -1 prior to infection. Twenty-four hours after the last injection (day 0), mice were intraperitoneally infected with a dose of 5 × 10^7^ CFU of type 2 *S. suis* wild-type strain P1/7. Non-infected mice were used as negative controls for basal cytokine expression. Plasma was collected 24 h post-infection and production of cytokines and chemokines was measured by ELISA. Data represent mean values (in ng/ml) ± SEM (*n* = 20, results combined from two experiments). ^*^P < 0.05 or ^**^P < 0.01, indicates statistically significant differences between mock-treated (−) and PK-136-treated (+) mice infected with *S. suis*.

It was previously demonstrated that an exaggerated inflammatory response leads to pathological consequences during the acute phase of *S. suis* infection (Dominguez-Punaro Mde et al., 2008). Thus, to provide more insight into the possible deleterious role of NK1.1^+^ cells during *S. suis* infection, we measured plasma levels of several cytokines and chemokines following *S. suis* infection after 24 h post-infection. As shown in Figure 5, after infection, NK1.1^+^ cell depleted-mice exhibited significantly reduced levels of the cytokines IL-6, IL-12p40, and G-CSF, and of the chemokines CCL2 and CCL5 compared to mock-treated infected mice (*P* < 0.05). No significant plasma levels of IFN-γ or TNF-α were observed under these experimental conditions and evaluated time point (data not shown), as previously reported (Dominguez-Punaro Mde et al., 2008).

Altogether, despite NK cells are the main producer of IFN-γ during early steps of GBS infection, these cells may have a limited role in the pathogenesis of the acute phase of GBS infection and/or other cells compensate for the absence of NK cells (Berg et al., 2005). In contrast, NK cells seem to be involved in the amplification of the inflammatory response during *S. suis* infection and can contribute to the exaggerated response early after infection.

### DCs are required for IFN-γ production by NK cells in response to encapsulated streptococci

**Figure 6:**
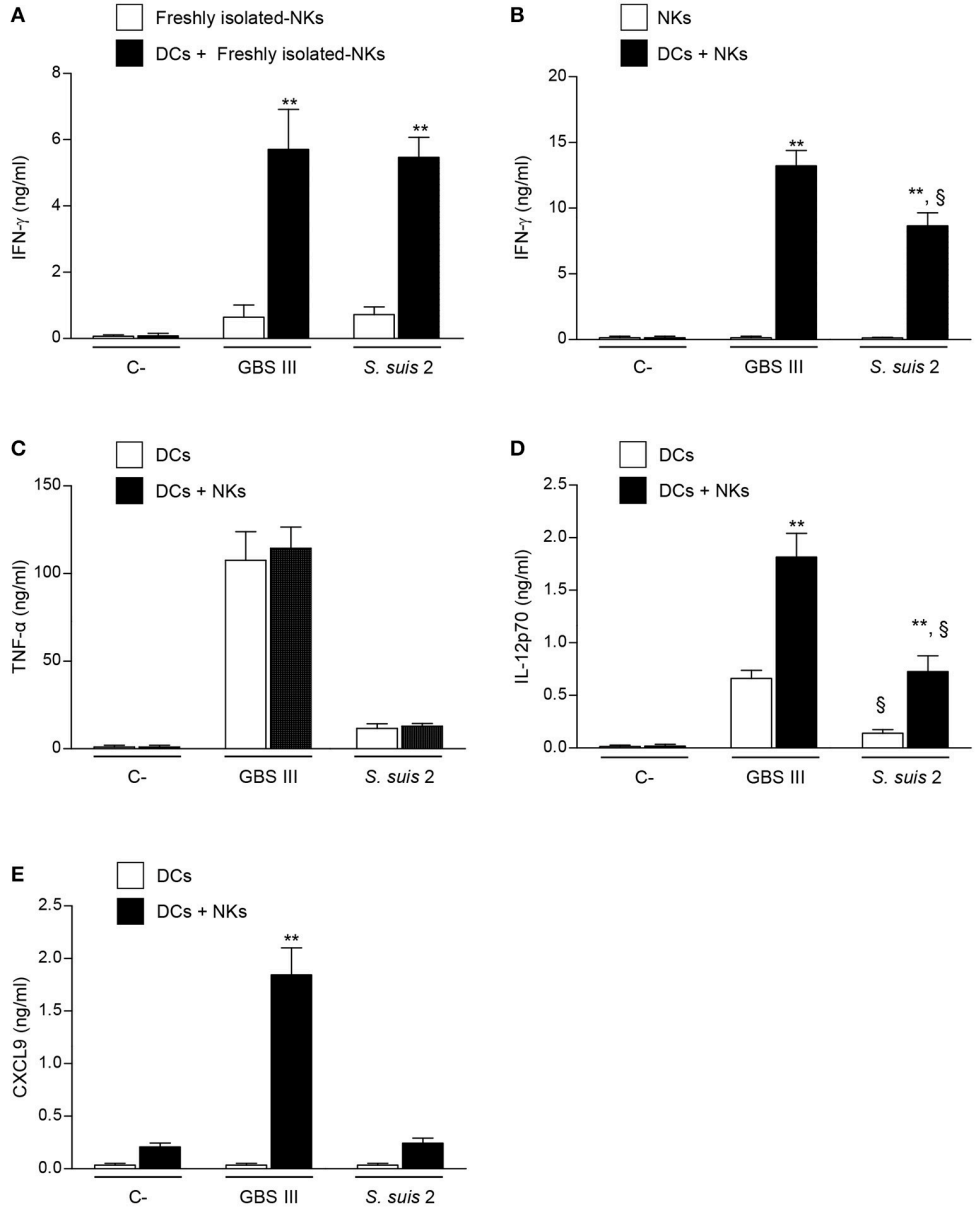
Cytokine profiles of DC-NK cell co-cultures in contact with GBS or *S. suis*. DCs and NK cells (at a ratio of 1:3) were stimulated with type III GBS wild-type strain COH-1 or type 2 *S. suis* wild-type strain P1/7 (2.5 × 10^5^ CFU, initial DC-bacteria MOI:1). After bacterium-cell contact, antibiotics were added to prevent cell toxicity. Supernatants were collected at 14 h of incubation, and IFN-γ **(A,B)**, TNF-α **(C)**, IL-12p70 **(D)**, and CXCL9 **(E)** levels were measured by ELISA. Non-stimulated cells (medium alone) served as negative (C-) controls. In addition, single-DC or NK cell cultures were also included as controls. Data represent mean values (in ng/ml) ± SEM of eight distinct experiments. ^**^P < 0.01, indicates statistically significant differences between single cell cultures vs co-cultures. ^§^*P* < 0.01, indicates statistically significant differences between GBS-infected cells vs. *S. suis*-infected cells. Unless otherwise indicated NKs states for culture-expanded splenic NK cells.

The fact that TLRs have been discovered to be expressed by NK cells has opened a new interest in their putative involvement in the innate immune response to bacterial infections (Adib-Conquy et al., 2014). Some studies reported a direct interaction of bacteria or bacterial components with NK cells (Schmidt et al., 2004; Yun et al., 2005; Esin et al., 2008; Marcenaro et al., 2008; Mian et al., 2010; Adib-Conquy et al., 2014). To evaluate the possibility that NK cells can be directly activated by live encapsulated streptococci, we incubated freshly isolated-NK cells from naïve mice with type III GBS or type 2 *S. suis*. As displayed in Figure 6A, NK cells produced low amounts of IFN-γ after direct contact with these two pathogenic streptococci. Freshly, MACS-purified splenic NK cells might contain contamination with other immune cells, such as APCs. Thus, to increase purity and yield, we used an *in vitro* expanded culture of splenic NK cells supplemented with IL-2 to confirm the obtained data. The purity of expanded NK cells was higher than 93% of NK1.1^+^CD3^–^ cells, and more importantly, did not contain contaminating NK1.1^–^ APCs (Figure S1). Using this highly pure culture of NK cells, production of IFN-γ after direct contact with either GBS or *S. suis* was not significantly different from non-infected control cells (Figure 6B). Similar data were obtained when other cytokines were tested or when different bacterial MOIs were used (data not shown).

To assess the importance of accessory cells in the activation of NK cells by type III GBS or type 2 *S. suis*, NK cells were co-cultured with DCs, as a source of APCs. A marked increase in IFN-γ production was observed in infected DC-NK cell co-cultures compared to non-infected counterparts or compared to infected-NK cell single cultures. This increase was observed when using either freshly isolated- or expanded-NK cells (Figures 6A,B), and was dependent on the number of NK cells in the co-culture (Figure S2). It should be noted that single cell cultures of DCs did not produce IFN-γ upon infection (data not shown). These data indicate that DCs are required to induce IFN-γ production by NK cells in the context of encapsulated streptococcal infections. In general, and similarly to that observed *ex vivo*, type III GBS showed higher capacity to induce *in vitro* IFN-γ production by NK cells in co-culture than type 2 *S. suis* (Figure S2).

### NK cells provide a positive feedback loop for DC activation which differs between GBS and *S. suis*

Because the crosstalk between DCs and NK cells is bidirectional (Moretta et al., 2006; Barreira da Silva and Munz, 2011; Souza-Fonseca-Guimaraes et al., 2012), it was important to determine whether the production of IFN-γ influences the magnitude and profile of cytokine secretion by DC-NK cell co-cultures. Type III GBS induced a high production of TNF-α by DCs and this production was unchanged in DC-NK cell co-cultures (Figure 6C). Thus, and similarly to that observed *ex vivo* (Figure 2A), NK cells did not produce TNF-α and did not influence the production of this cytokine by infected DCs. A similar pattern was observed for type 2 *S. suis*-infected co-cultures; however, levels of TNF-α secretion by DCs were markedly lower than those observed for type III GBS (Figure 6C). Similar results were obtained when the pro-inflammatory cytokine IL-6 was evaluated with either GBS or *S. suis* (data not shown). In contrast, production of IL-12p70 in infected DC-NK cell co-cultures was significantly higher than infected-DC single cultures for both pathogens (*P* < 0.01; Figure 6D). Yet, type 2 *S. suis* induced lower levels of IL-12p70 by either DCs or DC-NK cell co-cultures than those obtained upon infection with type III GBS (*P* < 0.01; Figure 6D). Finally, the production of CXCL9 in DC-NK cell co-cultures was evaluated as it is a chemokine known to be induced by IFN-γ (Robertson, 2002). Type III GBS failed to induce this CXCL9 production by DCs in single cultures, but its production was markedly increased in co-cultures (*P* < 0.01; Figure 6E), indicating an important positive feedback loop of NK cells (probably via IFN-γ) for this chemokine production. In contrast to GBS, neither DC single cultures nor DC-NK cell co-cultures produced significant levels of CXCL9 in contact with type 2 *S. suis* (Figure 6E), suggesting that the synergistic effect between DCs and NK cells differs in magnitude and/or profile upon the streptococcal species.

### Cell-cell contact and soluble factors are important in the DC-NK cell crosstalk

**Figure 7:**
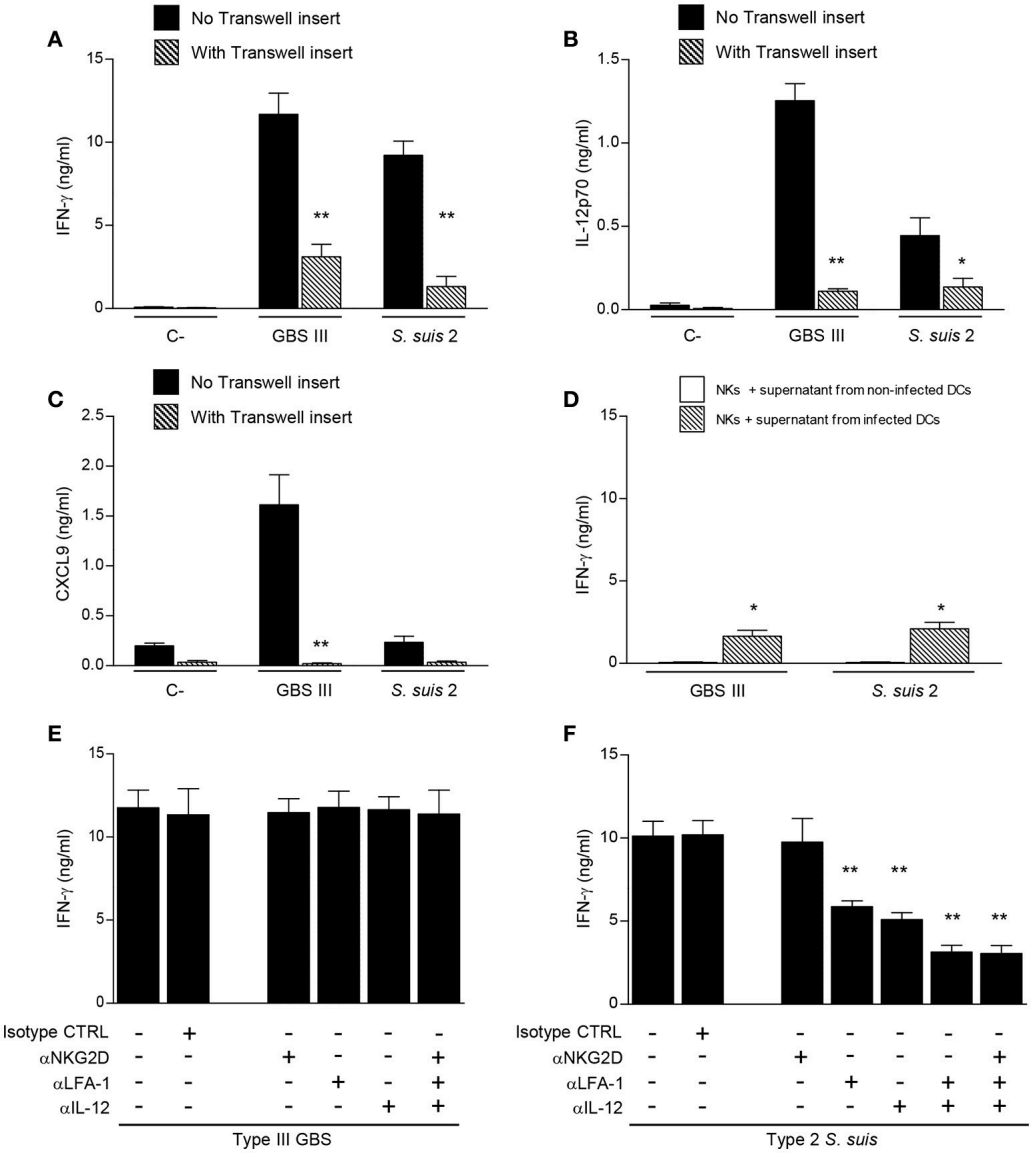
Different mechanisms mediate DC-NK cell crosstalk during GBS or *S. suis* infections. DCs and culture-expanded splenic NK cells were co-cultured (at a ratio 1:3) in the presence or not of a Transwell insert **(A–C)** or pre-incubated with neutralizing antibodies (anti-IL-12, 20 μg/ml; anti-NKG2D, 10 μg/ml; and/or anti-LFA-1, 10 μg/ml) or with the isotype controls (CTRL) rat IgG2a and Armenian hamster IgG **(E,F)**. Co-cultures were stimulated with type III GBS wild-type strain COH-1 or type 2 *S. suis* wild-type strain P1/7 (2.5 × 10^5^ CFU, initial DC-bacteria MOI:1). After bacterium-cell contact, antibiotics were added to prevent cell toxicity. In **(D)** NK cells alone were incubated with supernatants from GBS or *S. suis*-infected DCs. After 14 h of incubation, IFN-γ **(A, D–F)**, IL-12p70 **(B)** and CXCL9 **(C)** levels were measured by ELISA. Non-stimulated cells (medium alone or supernatant from non-infected DCs) served as negative (C-) controls. Data represent mean values (in ng/ml) ± SEM of eight distinct experiments. ^*^P < 0.05 or ^**^P < 0.01, indicates statistically significant differences compared to respective non-treated or mock-treated controls in each graph.

To further characterize the interactions between DCs and NK cells during encapsulated streptococcal infections, the requirement of cell-cell contact was evaluated using a Transwell® system. As shown in Figure 7A, the absence of direct cell-cell contact resulted in significant inhibition of IFN-γ production by NK cells for either GBS or *S. suis*-infected co-cultures (*P* < 0.01). Despite this strong inhibition, IFN-γ production was still higher than that observed in respective non-infected controls (*P* < 0.05), indicating that part of this production is independent of cell-cell contact and thus probably mediated by soluble factors. Cell-cell contact was also markedly required for the synergistic effect on cytokine production observed in infected DC-NK cell co-cultures (Figures 7B,C). Indeed, the increased production of IL-12p70 and CXCL9 in type III GBS-infected co-cultures was almost completely inhibited (*P* < 0.01), indicating that this synergistic effect mostly depends on cell-cell contact. Similarly, a marked but yet not complete inhibition was observed in the IL-12p70 production by *S. suis*-infected co-cultures when using a Transwell® insert (Figure 7B).

To confirm that soluble factors might play a minor, but still significant, role in NK-derived IFN-γ production, NK cells alone were incubated with supernatants from either GBS or *S. suis*-infected DCs. Production of IFN-γ by NK cells was significantly higher after incubation with supernatants from infected DCs than that obtained with supernatants from non-infected DCs (Figure 7D). This production was similar to the observed residual IFN-γ production in co-cultures containing a Transwell® insert (Figure 7A).

Thus, for both GBS and *S. suis*, the IFN-γ production by NK cells and the synergistic effect on cytokine production by DC-NK cell co-cultures are principally dependent on cell-cell contact, yet a contribution of soluble factors to the IFN-γ production is observed.

### The molecular components involved in the DC-NK cell crosstalk differ upon the streptococcal species

Because cell-cell contact is important in the IFN-γ production by NK cells in the presence of DCs, we were interested in characterizing the molecular components involved in this interaction. We focused on the LFA-1 adhesion molecule and on the NKG2D-activating receptor, as these two receptors have been reported to play a role in the DC-NK crosstalk or during bacterial infections (Binnerts and van Kooyk, 1999; Guan et al., 2007; Wesselkamper et al., 2008; Jiao et al., 2011; Bouwer et al., 2013). When DC-NK cell co-cultures were pre-treated with neutralizing antibodies against LFA-1 or NKG2D, either alone or in combination, IFN-γ production was unaltered after type III GBS stimulation (Figure 7E). Similarly, neutralization of NKG2D did not affect IFN-γ production by NK cells upon type 2 *S. suis* stimulation (Figure 7F). However, and in contrast to that observed for GBS, neutralization of LFA-1 significantly reduced IFN-γ secretion in *S. suis*-infected co-cultures (*P* < 0.01).

It has been shown that IL-12 is required for IFN-γ production, and the effect of IL-12 is most eminent in combination with IL-18, for both NK and T cells (Okamura et al., 1998). Since IL-18 is not produced by GBS- or *S. suis*-infected DCs under our culture conditions (data not shown), we investigated more specifically the role of IL-12 in the IFN-γ production induced by these two pathogens. Anti-IL-12 blocking mAb did not affect IFN-γ production by NK cells in contact with type III GBS-infected DCs, while its production was significantly reduced in DC-NK cell co-cultures infected with type 2 *S. suis* (Figures 7E,F). Combination of anti-LFA-1, anti-NKG2D or anti-IL-12 neutralizing antibodies, either in pairs or altogether, confirmed that LFA-1 and IL-12 have a synergistic effect on *S. suis*-induced IFN-γ production by NK cells (Figure 7F). Indeed a decrease of 70% in IFN-γ production was observed in co-cultures treated with a combination of anti-LFA-1 and anti-IL-12 neutralizing antibodies compared to non-treated controls (*P* < 0.01). Absence of effect on IFN-γ production by the isotype controls confirmed the specificity of the inhibition. In contrast, the combination of neutralizing antibodies against the three molecules did not affect IFN-γ production in GBS-infected DC-NK cell co-cultures (Figure 7E).

### *S. suis* CPS but not GBS CPS impairs NK cell activation

**Figure 8:**
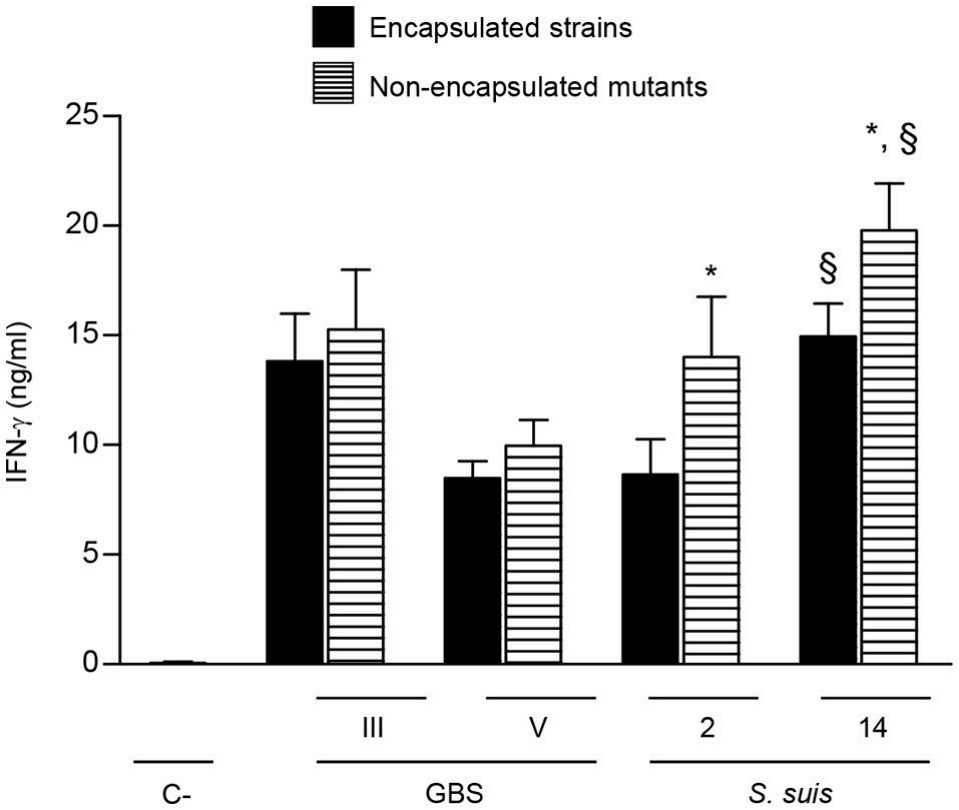
Role of bacterial capsular polysaccharide in the modulation of cytokine production by NK cells. DC and NK cell co-cultures (at a ratio 1:3) were stimulated with type III or type V GBS wild-type strains type 2 or type 14 *S. suis* wild-type strains or their respective non-encapsulated mutant strains (2.5 × 10^5^ CFU, initial DC-bacteria MOI:1). After bacterium-cell contact, antibiotics were added to prevent cell toxicity. Supernatants were collected at 14 h of incubation and IFN-γ levels were measured by ELISA. Non-stimulated cells (medium alone) served as negative (C-) controls. Data represent mean values (in ng/ml) ± SEM of five distinct experiments. ^*^P < 0.05, indicates statistically significant differences between wild-type strains and their respective non-encapsulated mutants. ^§^*P* < 0.01, indicates statistically significant differences between type 2 *S. suis* and type 14 *S. suis* strains.

The CPS is an important virulence factor contributing to the pathogenesis of disease caused by GBS or *S. suis*. However, the presence of CPS differently modulates the interactions of these bacteria with immune cells (Maisey et al., 2008; Lecours et al., 2011, 2012; Fittipaldi et al., 2012; Lemire et al., 2012a,b, 2014; Segura, 2012; Roy et al., 2015). To dissect the impact of bacterial CPS on NK cell activation, two different serotypes harboring distinct CPS structures and their respective non-encapsulated mutants were evaluated for each bacterial species. NK cell production of IFN-γ induced by encapsulated type V GBS was slightly lower than that observed with type III GBS. Nevertheless, the presence of CPS did not significantly affect NK cell activation by either of GBS serotypes (Figure 8). In the case of *S. suis*, type 14 *S. suis* induced higher levels of IFN-γ secretion by NK cells that type 2 *S. suis* (*P* < 0.01; Figure 8). When non-encapsulated mutants were tested, both induced significantly higher levels IFN-γ production than their respective wild-type strains (*P* < 0.05). In agreement with their enhanced capacity to stimulate IFN-γ release by NK cells, non-encapsulated mutants of either type 2 or type 14 *S. suis* induced higher production of several cytokines by single DC cultures that the wild-type strains (Figure S6). Albeit differences in IFN-γ levels induced by different serotypes, probably related to the intrinsic capacity of each strain to activate cells, the bacterial CPS effect on NK activation was species-dependent but serotype-independent.

## Discussion

Despite advances in understanding the pathogenesis of disease caused by encapsulated bacteria, more specifically on the interactions between these bacteria and cells of the innate immunity, such as DCs, macrophages and neutrophils, few studies had focused on the importance of NK cells during GBS or *S. suis* infections. Furthermore, a convincing analysis at single cell level of the source(s) of IFN-γ in the early immune response to an acute infection with these bacterial pathogens is still missing. In the present work, we addressed for the first time the involvement of splenic NK cells in the innate immune response to GBS and *S. suis* and the role of DCs in initiating NK cell activation.

A critical role for IFN-γ in the defense against a variety of diseases, including extracellular bacterial infections, has been evidenced (Bouwer et al., 2013; Adib-Conquy et al., 2014). Several lymphocyte subsets, including NK cells, NKT cells, and T cells are capable of producing IFN-γ. In this study, during the acute phase of systemic GBS or *S. suis* infection, NK cells were identified as the main producers of IFN-γ. However, comparatively to GBS, this dominance disappears rapidly during *S. suis* infection. In fact, GBS infection results in a stronger boost of early IFN-γ production with concomitant higher activation of the innate immune cascade than *S. suis*. Lachance et al. reported that a hyper-virulent type 2 *S. suis* strain, responsible of a deadly human outbreak in China, has evolved to massively activate IFN-γ production leading to a rapid and lethal streptococcal toxic shock-like syndrome. *S. suis* European strains (like the one used in the present study) have been reported to be less virulent than the Chinese epidemic strain, and microarray analyses indicated lower induction of IFN-γ gene expression in European strain-infected mice than Chinese-strain infected counterparts (Lachance et al., 2013a). Our data confirm a weak IFN-γ response induced by an European-origin type 2 *S. suis* strain. Yet, the strain is able to induce clinical signs of sepsis, meningitis, and death, suggesting that an inflammatory response is indeed induced, probably involving other innate immune cells and cytokines. In this regard, TNF-α and CD69 expression was observed in splenic cells from *S. suis*-infected animals.

NK cells have been identified in several studies as the main early source of IFN-γ during infection by several bacterial pathogens, most of them intracellular organisms (Souza-Fonseca-Guimaraes et al., 2012; Adib-Conquy et al., 2014). In the case of extracellular pathogens fewer studies are available; though, NK cells were reported to be the dominant source of IFN-γ in brains or lungs during pneumococcal infections (McNeela et al., 2010; Mitchell et al., 2012). Other innate cellular sources of IFN-γ are NKT cells, which were shown in this study to be the second source of IFN-γ during acute systemic infections with GBS or, to a lesser extent, *S. suis*. In agreement with these findings, a previous study reported that NKT cells recognize diacylglycerol-containing glycolipids from GBS. However, the same study reported that NKT cells fail in responding to glycolipids from *S. suis* serotype 1 *in vitro* (Kinjo et al., 2011). Albeit at very low levels, our *in vivo* work shows that NKT cells might be activated during type 2 *S. suis* infection. It has been proposed that NKT cells can be activated by cytokines, particularly IL-12, even in the absence of CD1d antigen presentation (Brigl et al., 2011). As *S. suis*-activated DCs produce IL-12, a certain level of cytokine-driven activation of NKT cells might occurs during *S. suis* infection. Another possibility is that the strain used in this study, which belongs to serotype 2, does contain CD1d binding antigens. Further studies are required to address the role of NKT cells during these two streptococcal infections and the contribution of CD1d-dependent *vs*. CD1d-independent activation.

Interestingly, for both *S. suis* and GBS, NK cells do not seem to contribute to TNF-α production. In contrast, TNF-α production by NK cells was reported in response to *Escherichia coli* infection (Gur et al., 2013), and in an influenza virus and *Staphylococcus aureus* co-infection mouse model (Small et al., 2010). GBS and *S. suis* have been reported to induce high levels of TNF-α by DCs, macrophages and other host cells, which indicates that these bacteria possess the capacity to induce this cytokine release (Maisey et al., 2008; Lecours et al., 2011, 2012; Fittipaldi et al., 2012; Lemire et al., 2012b, 2014). Few studies are available on CD69 expression by NK cells during *in vivo* bacterial infections, but up-regulation of this molecule have been shown on NK cells activated *in vitro* with purified bacterial pathogen-associated molecular patterns (PAMPs) (Schmidt et al., 2004; Tsujimoto et al., 2005; Mian et al., 2010). Studies using non-infectious models suggest that CD69 up-regulation seems to prepare NK cells for further responses, such as cytotoxicity or cytokine production (Clausen et al., 2003; Sancho et al., 2005). Interestingly, in our bacterial infection models, we also observed a decrease in NK1.1^+^ cells in the spleens, which might suggests NK cell trafficking upon activation toward the sites of bacterial infection. On the other hand, NK cell death induced by *E. coli* infection has been reported (Gur et al., 2013). More investigations are necessary to understand NK1.1^+^ cell lifespan and trafficking during GBS or *S. suis* infections.

NK cells display both beneficial and deleterious effects, depending on the circumstances. Indeed, NK cells are closely associated with both the fight against bacterial infection and the damages associated with an excessive inflammatory response. In different models, NK cells have been shown to be protective by limiting bacteremia levels (Nilsson et al., 1999; Small et al., 2008; Souza-Fonseca-Guimaraes et al., 2012). Deleterious effects of NK cell activation were also reported after infection with both gram-negative bacteria (Badgwell et al., 2002) and gram-positive bacteria (Kerr et al., 2005; Christaki et al., 2015). In this study, depletion of NK1.1^+^ cells did not alter the outcome of GBS infection, suggesting a redundant role of other immune cells. In contrast to GBS, but similarly to *Streptococcus pyogenes* (Goldmann et al., 2005), NK1.1^+^ cells play a deleterious role early during an infection with *S. suis* which is associated with amplification of the inflammatory response rather than the control of bacteremia levels (Goldmann et al., 2005). In the case of *Streptococcus pneumoniae*, NK cells induce higher bacteremia levels and concomitant increased inflammatory response (Badgwell et al., 2002; Small et al., 2008). Thus, NK1.1^+^ cells seem to differently affect the pathogenesis of the infections caused by distinct encapsulated streptococci.

Some studies have addressed the role of direct interaction of pathogens or pathogen-derived ligands in NK cell activation. For example, it has been demonstrated that NK cells directly recognize bacterial PAMPs, such as TLR ligands, which triggers IFN-γ and CD69 expression among other pathways (Tsujimoto et al., 2005; Esin et al., 2008; Marcenaro et al., 2008; Souza-Fonseca-Guimaraes et al., 2012). GBS and *S. suis* have been shown to possess ligands for TLR2 and to activate innate immune cells via several TLRs (Wennekamp and Henneke, 2008; Lecours et al., 2012; Lemire et al., 2014). Yet, in our study, GBS or *S. suis* failed to directly activate NK cells *in vitro*. In fact, indirect activation of NK cells by bacteria is increasingly studied and the requirement of accessory cells, such as DCs and macrophages, as key partners for NK cell activation has been widely documented mainly because they are a major source of IL-12, and they can also produce IL-15, IL-18, and IFN-α (Newman and Riley, 2007; Humann and Lenz, 2010; Barreira da Silva and Munz, 2011; Adib-Conquy et al., 2014). Rosati et al. showed NK cell activity in total spleen cells after type Ia GBS infection (Rosati et al., 1998). It was indirectly suggested that IL-12 released by macrophages could activate NK cells. An early study showed that peritoneal macrophages and bone marrow-derived cells from severe combined immunodeficiency mice cultured separately with type III GBS fail to produce IFN-γ, whereas co-cultures do produce IFN-γ. These cultures contained around 40% of NK1.1^+^ cells (Derrico and Goodrum, 1996). By using a refined co-culture system of DCs and NK cells, we demonstrated that GBS or *S. suis*-infected DCs are important inducers of IFN-γ production by NK cells. Only few studies have addressed the modulation of the DC-NK cell crosstalk by streptococci. *In vitro* studies showed that this crosstalk is primarily responsible for IFN-γ release induced by *S. pneumoniae* (Elhaik-Goldman et al., 2011) or *Streptococcus salivarius* (Bouwer et al., 2013). Cell contact and cytokine release into the synapse between DCs and NK cells may enhance their crosstalk (Borg et al., 2004). The underlying mechanisms have been studied mainly in the context of intracellular bacterial infections and seem to differ upon the pathogen; being, for example, either NKG2D receptor-dependent (Borchers et al., 2006) or -independent (Lapaque et al., 2009). Data from this study demonstrated that cell-cell contact between DCs and NK cells is critical for the IFN-γ production in response to GBS or *S. suis*. Interestingly, in the context of *S. suis* infection, the contact dependency was in part mediated by LFA-1. Yet, this mechanism may not be generalized to all streptococcal species as modulation of the DC-NK cell crosstalk by GBS or *S. salivarus* (Bouwer et al., 2013) is not dependent on LFA-1. In this study, a direct involvement of NKG2D was not evidenced after infection with either GBS or *S. suis*, as likewise reported for *S. salivarus* infection (Bouwer et al., 2013). On the other hand, DCs, in contact with GBS or *S. suis*, produce several cytokines and chemokines, notably IL-12 (Lecours et al., 2011, 2012; Lemire et al., 2012a,b, 2014) and thus have the potential to activate NK cells. Their contribution was minor, yet significant. IL-12 was identified as one of the soluble mediators required for an optimal IFN-γ production by NK cells in the context of *S. suis* infection, as already reported for *Listeria monocytogenes* (Humann and Lenz, 2010) or *Salmonella* (Lapaque et al., 2009). Yet, unlike *S. suis*, several cytokines or redundant pathways might be involved in GBS modulation of the DC-NK cell interactions. Activation of NK cells by cytokine trans-presentation, mainly IL-15, has been previously reported (Lucas et al., 2007; Mortier et al., 2008; Bihl et al., 2010; Zanoni et al., 2013) and cannot be ruled out as a possible mechanism in the context of GBS or *S. suis* infection.

It was further showed that NK cell activation had a synergistic effect in inducing cytokines and chemokines production by DCs in response to GBS or *S. suis*, notably by the increasing production of IL-12 mainly in a cell-cell contact dependent manner. Interestingly, DC production of the chemokine CXCL9 in contact with type III GBS was completely dependent on a synergistic effect between DCs and NK cells. Yet, this synergy is not observed in *S. suis*-infected co-cultures, suggesting once again that modulation of the DC-NK cell crosstalk varies upon the pathogen. Resting human NK cells have been found to migrate in response to known ligands for CXC chemokine receptor (CXCR) 3, like CXCL9 and CXCL10 (Robertson, 2002; Maghazachi, 2010). It might be suggested that activated NK cells can modulate DCs to produce specific chemokines to attract other cells.

GBS and *S. suis* are the sole Gram-positive bacteria harboring a side chain terminated by sialic acid in their CPS compositions. In spite of this and other CPS biochemical and structural similarities, GBS and *S. suis* pathogenic mechanisms and interplay with components of the immune system, including DCs, seem to radically differ (Lecours et al., 2011, 2012; Lemire et al., 2012a,b, 2014; Roy et al., 2015). In agreement with these previous studies, GBS CPS does not seem to impair DC-NK cell crosstalk, and confirm the fact that this bacterial CPS is not acting as a physical barrier impairing PAMP ligation to activating receptors on DCs. In marked contrast, *S. suis* CPS readily acts as a cloaking factor and impairs optimal activation of the DC-NK cell interactions. This CPS immuno-modulatory effect might explain, at least in part, the lower inflammatory cascade induced *in vivo* by a *S. suis* infection when compared to GBS-infected mice. In spite of biochemical differences in CPS structures, similar behaviors were observed when other GBS or *S. suis* serotypes were evaluated. Type III or type V GBS CPS does not significantly impair DC activation and consequently the DC-NK crosstalk, whereas both, type 2 and type 14 *S. suis* CPSs hinder not only optimal DC functions (Lecours et al., 2011, 2012; Lemire et al., 2014) but also the close interactions between these cells and NK cells. These findings suggest that the impact of bacterial CPSs on NK cell activation varies upon the streptococcal species but does not seem to depend on the serotype. It would have been interesting to study the role of the CPS in the *in vivo* context. However, the non-encapsulated mutants are rapidly cleared by the immune system and fail to induce infection in mice. To the best of our knowledge, no study has addressed the role of the streptococcal CPS in NK cell activation.

Our results suggest that NK cells and IFN-γ play an active role in the orchestration of the innate immune response during the acute phase of encapsulated streptococcal infections. NK1.1^+^ cells, by amplifying the inflammatory response, contribute to the progression of *S. suis* infection but seems to have a redundant role during GBS infection. Based on our findings, we propose a model where DCs first recognize GBS or *S. suis* and produce several cytokines which promote IFN-γ induction in NK cells and enhance the interactions and the synergistic effect between DCs and NK cells. The close contact between these cells is essential for a fully NK cell activation; however, GBS and *S. suis* differently modulate this interaction. Overall this study suggests that the molecular pathways governing the DC-NK cell crosstalk differ upon the pathogen and should not be generalized when studying bacterial infections.

## Author contributions

Conceived and designed the experiments: PL and MS. Performed the experiments: PL and TG. Analyzed the data: PL, TG, JT, and MS. Contributed to the writing of the manuscript: PL, TG, JT, and MS. All the authors read and approved the final manuscript.

### Conflict of interest statement

The authors declare that the research was conducted in the absence of any commercial or financial relationships that could be construed as a potential conflict of interest.

## Abbreviations

APCs: antigen-presenting cells
CCL: C-C motif chemokine ligand
CPS: capsular polysaccharide
CXCL: C-X-C motif chemokine ligand
DC: dendritic cell
GBS: Group B *Streptococcus*
GM-CSF: granulocyte macrophage colony stimulating factor
G-CSF: granulocyte-colony stimulating factor
IFN: interferon
IL: interleukin
i.p.: intraperitoneally
mAb: monoclonal antibody
MACS: magnetically-activated cell sorting
MOI: multiplicity of infection
NK: natural killer
PAMP: pathogen-associated molecular pattern
THB: Todd-Hewitt broth
THA: Todd-Hewitt agar
TLR: Toll-like receptor
TNF: tumor necrosis factor.

## References

[B1] Adib-Conquy M. Scott-Algara D. Cavaillon J. M. Souza-Fonseca-Guimaraes F. (2014). TLR-mediated activation of NK cells and their role in bacterial/viral immune responses in mammals. Immunol. Cell Biol. 92, 256–262. 10.1038/icb.2013.99 24366517

[B2] Badgwell B. Parihar R. Magro C. Dierksheide J. Russo T. Carson W. E. (2002). Natural killer cells contribute to the lethality of a murine model of *Escherichia coli* infection. Surgery 132, 205–212. 10.1067/msy.2002.125311 12219013

[B3] Barreira da Silva R. Munz C. (2011). NK cell activation by dendritic cells: balancing inhibitory and activating signals. Cell Mol. Life Sci. 68, 3505–3518. 10.1007/s00018-011-0801-8 21861182 PMC11114903

[B4] Benigni G. Dimitrova P. Antonangeli F. Sanseviero E. Milanova V. Blom A.. (2017). CXCR3/CXCL10 axis regulates neutrophil-NK cell cross-talk determining the severity of experimental osteoarthritis. J. Immunol. 198, 2115–2124. 10.4049/jimmunol.1601359 28108560

[B5] Berg R. E. Crossley E. Murray S. Forman J. (2005). Relative contributions of NK and CD8 T cells to IFN-gamma mediated innate immune protection against *Listeria monocytogenes*. J. Immunol. 175, 1751–1757. 10.4049/jimmunol.175.3.1751 16034116 PMC1615713

[B6] Bihl F. Pecheur J. Breart B. Poupon G. Cazareth J. Julia V.. (2010). Primed antigen-specific CD4+ T cells are required for NK cell activation *in vivo* upon *Leishmania major* infection. J. Immunol. 185, 2174–2181. 10.4049/jimmunol.1001486 20624944

[B7] Binnerts M. E. van Kooyk Y. (1999). How LFA-1 binds to different ligands. Immunol. Today 20, 240–245. 10322304 10.1016/s0167-5699(99)01467-x

[B8] Borchers M. T. Harris N. L. Wesselkamper S. C. Zhang S. Chen Y. Young L.. (2006). The NKG2D-activating receptor mediates pulmonary clearance of *Pseudomonas aeruginosa*. Infect. Immun. 74, 2578–2586. 10.1128/IAI.74.5.2578-2586.2006 16622193 PMC1459711

[B9] Borg C. Jalil A. Laderach D. Maruyama K. Wakasugi H. Charrier S.. (2004). NK cell activation by dendritic cells requires the formation of a synapse leading to IL-12 polarization in dendritic cells. Blood 104, 3267–3275. 10.1182/blood-2004-01-0380 15242871

[B10] Bouwer A. L. Saunderson S. C. Dunn A. C. Lester K. L. Crowley L. R. Jack R. W.. (2013). Rapid IFN-gamma release from NK cells induced by a streptococcal commensal. J. Interferon Cytokine Res. 33, 459–466. 10.1089/jir.2012.0116 23659669

[B11] Brigl M. Tatituri R. V. Watts G. F. Bhowruth V. Leadbetter E. A. Barton N.. (2011). Innate and cytokine-driven signals, rather than microbial antigens, dominate in NKT cell activation during microbial infection. J. Exp. Med. 208, 1163–1177. 10.1084/jem.20102555 21555485 PMC3173255

[B12] Calzas C. Lemire P. Auray G. Gerdts V. Gottschalk M. Segura M. (2015). Antibody response specific to the capsular polysaccharide is impaired in *Streptococcus suis* serotype 2-infected animals. Infect. Immun. 83, 441–453. 10.1128/IAI.02427-14 25385801 PMC4288899

[B13] Christaki E. Diza E. Giamarellos-Bourboulis E. J. Papadopoulou N. Pistiki A. Droggiti D. I.. (2015). NK and NKT Cell depletion alters the outcome of experimental pneumococcal pneumonia: relationship with regulation of IFN-gamma production. J. Immunol. Res. 2015:532717. 10.1155/2015/532717 26114123 PMC4465773

[B14] Cieslewicz M. J. Chaffin D. Glusman G. Kasper D. Madan A. Rodrigues S.. (2005). Structural and genetic diversity of Group B *Streptococcus* capsular polysaccharides. Infect. Immun. 73, 3096–3103. 10.1128/IAI.73.5.3096-3103.2005 15845517 PMC1087335

[B15] Clarke D. Letendre C. Lecours M. P. Lemire P. Galbas T. Thibodeau J.. (2016). Group B *Streptococcus* induces a robust IFN-gamma response by CD4(+) T cells in an *in vitro* and *in vivo* model. J. Immunol. Res. 2016:5290604. 10.1155/2016/5290604 26989699 PMC4771917

[B16] Clausen J. Vergeiner B. Enk M. Petzer A. L. Gastl G. Gunsilius E. (2003). Functional significance of the activation-associated receptors CD25 and CD69 on human NK cells and NKT cells. Immunobiology 207, 85–93. 10.1078/0171-2985-00219 12675266

[B17] Cusumano V. Mancuso G. Genovese F. Delfino D. Beninati C. Losi E.. (1996). Role of gamma interferon in a neonatal mouse model of Group B streptococcal disease. Infect. Immun. 64, 2941–2944. 8757817 10.1128/iai.64.8.2941-2944.1996 PMC174171

[B18] Cusumano V. Midiri A. Cusumano V. V. Bellantoni A. De Sossi G. Teti G.. (2004). Interleukin-18 is an essential element in host resistance to experimental Group B streptococcal disease in neonates. Infect. Immun. 72, 295–300. 10.1128/IAI.72.1.295-300.2004 14688108 PMC344002

[B19] Derrico C. A. Goodrum K. J. (1996). IL-12 and TNF-alpha mediate innate production of gamma interferon by Group B *Streptococcus*-treated splenocytes of severe combined immunodeficiency mice. Infect. Immun. 64, 1314–1320. 8606095 10.1128/iai.64.4.1314-1320.1996 PMC173920

[B20] Dominguez-Punaro Mde L. Segura M. Radzioch D. Rivest S. Gottschalk M. (2008). Comparison of the susceptibilities of C57BL/6 and A/J mouse strains to *Streptococcus suis* serotype 2 infection. Infect. Immun. 76, 3901–3910. 10.1128/IAI.00350-08 18573893 PMC2519407

[B21] Elhaik-Goldman S. Kafka D. Yossef R. Hadad U. Elkabets M. Vallon-Eberhard A.. (2011). The natural cytotoxicity receptor 1 contribution to early clearance of *Streptococcus pneumoniae* and to NK-macrophage crosstalk. PLoS ONE 6:e23472. 10.1371/journal.pone.0023472 21887255 PMC3161738

[B22] Esin S. Batoni G. Counoupas C. Stringaro A. Brancatisano F. L. Colone M.. (2008). Direct binding of human NK cell natural cytotoxicity receptor NKp44 to the surfaces of mycobacteria and other bacteria. Infect. Immun. 76, 1719–1727. 10.1128/IAI.00870-07 18212080 PMC2292874

[B23] Fittipaldi N. Segura M. Grenier D. Gottschalk M. (2012). Virulence factors involved in the pathogenesis of the infection caused by the swine pathogen and zoonotic agent *Streptococcus suis*. Future Microbiol. 7, 259–279. 10.2217/fmb.11.149 22324994

[B24] Frenkel D. Zhang F. Guirnalda P. Haynes C. Bockstal V. Radwanska M.. (2016). Trypanosoma brucei co-opts NK cells to kill splenic B2 B cells. PLoS Pathog. 12:e1005733. 10.1371/journal.ppat.1005733 27403737 PMC4942092

[B25] Goldmann O. Chhatwal G. S. Medina E. (2005). Contribution of NK cells to the pathogenesis of septic shock induced by *Streptococcus pyogenes* in mice. J. Infect. Dis. 191, 1280–1286. 10.1086/428501 15776374

[B26] Gottschalk M. Higgins R. Jacques M. Mittal K. R. Henrichsen J. (1989). Description of 14 new capsular types of *Streptococcus suis*. J. Clin. Microbiol. 27, 2633–2636. 2480359 10.1128/jcm.27.12.2633-2636.1989 PMC267098

[B27] Gottschalk M. Xu J. Calzas C. Segura M. (2010). *Streptococcus suis*: a new emerging or an old neglected zoonotic pathogen? Future Microbiol. 5, 371–391. 10.2217/fmb.10.2 20210549

[B28] Goyette-Desjardins G. Auger J. P. Xu J. Segura M. Gottschalk M. (2014). *Streptococcus suis*, an important pig pathogen and emerging zoonotic agent - an update on the worldwide distribution based on serotyping and sequence typing. Emerg. Microbes Infect. 3:e45. 10.1038/emi.2014.45 26038745 PMC4078792

[B29] Guan H. Moretto M. Bzik D. J. Gigley J. Khan I. A. (2007). NK cells enhance dendritic cell response against parasite antigens via NKG2D pathway. J. Immunol. 179, 590–596. 10.4049/jimmunol.179.1.590 17579080

[B30] Gur C. Coppenhagen-Glazer S. Rosenberg S. Yamin R. Enk J. Glasner A.. (2013). NK cell-mediated host defense against uropathogenic *Escherichia coli* is counteracted by bacterial hemolysinA-dependent killing of NK cells. Cell Host Microbe 14, 664–674. 10.1016/j.chom.2013.11.004 24331464 PMC3868942

[B31] Humann J. Lenz L. L. (2010). Activation of naive NK cells in response to *Listeria monocytogenes* requires IL-18 and contact with infected dendritic cells. J. Immunol. 184, 5172–5178. 10.4049/jimmunol.0903759 20351186 PMC2920760

[B32] Jiao L. Gao X. Joyee A. G. Zhao L. Qiu H. Yang M.. (2011). NK cells promote type 1 T cell immunity through modulating the function of dendritic cells during intracellular bacterial infection. J. Immunol. 187, 401–411. 10.4049/jimmunol.1002519 21642541

[B33] Kerr A. R. Kirkham L. A. Kadioglu A. Andrew P. W. Garside P. Thompson H.. (2005). Identification of a detrimental role for NK cells in pneumococcal pneumonia and sepsis in immunocompromised hosts. Microbes Infect. 7, 845–852. 10.1016/j.micinf.2005.02.011 15893495

[B34] Kinjo Y. Illarionov P. Vela J. L. Pei B. Girardi E. Li X.. (2011). Invariant NKT cells recognize glycolipids from pathogenic Gram-positive bacteria. Nat. Immunol. 12, 966–974. 10.1038/ni.2096 21892173 PMC3178673

[B35] Lachance C. Gottschalk M. Gerber P. P. Lemire P. Xu J. Segura M. (2013a). Exacerbated type II interferon response drives hypervirulence and toxic shock by an emergent epidemic strain of *Streptococcus suis*. Infect. Immun. 81, 1928–1939. 10.1128/IAI.01317-12 23509145 PMC3676015

[B36] Lachance C. Segura M. Gerber P. P. Xu J. Gottschalk M. (2013b). TLR2-independent host innate immune response against an epidemic strain of *Streptococcus suis* that causes a toxic shock-like syndrome in humans. PLoS ONE 8:e65031. 10.1371/journal.pone.0065031 23724118 PMC3665724

[B37] Lapaque N. Walzer T. Meresse S. Vivier E. Trowsdale J. (2009). Interactions between human NK cells and macrophages in response to *Salmonella* infection. J. Immunol. 182, 4339–4348. 10.4049/jimmunol.0803329 19299734

[B38] Le Doare K. Heath P. T. (2013). An overview of global Group B *Streptococcus* epidemiology. Vaccine 31, D7–D12. 10.1016/j.vaccine.2013.01.009 23973349

[B39] Lecours M. P. Gottschalk M. Houde M. Lemire P. Fittipaldi N. Segura M. (2011). Critical role for *Streptococcus suis* cell wall modifications and suilysin in resistance to complement-dependent killing by dendritic cells. J. Infect. Dis. 204, 919–929. 10.1093/infdis/jir415 21849289

[B40] Lecours M. P. Letendre C. Clarke D. Lemire P. Galbas T. Benoit-Biancamano M. O.. (2016). Immune-responsiveness of CD4+ T cells during *Streptococcus suis* serotype 2 infection. Sci. Rep. 6:38061. 10.1038/srep38061 27905502 PMC5131321

[B41] Lecours M. P. Segura M. Fittipaldi N. Rivest S. Gottschalk M. (2012). Immune receptors involved in *Streptococcus suis* recognition by dendritic cells. PLoS ONE 7:e44746. 10.1371/journal.pone.0044746 22984550 PMC3440357

[B42] Lemire P. Calzas C. Segura M. (2013). The NOD2 receptor does not play a major role in the pathogenesis of Group B *Streptococcus* in mice. Microb. Pathog. 65, 41–47. 10.1016/j.micpath.2013.09.006 24107312

[B43] Lemire P. Houde M. Segura M. (2012a). Encapsulated Group B *Streptococcus* modulates dendritic cell functions via lipid rafts and clathrin-mediated endocytosis. Cell. Microbiol. 14, 1707–1719. 10.1111/j.1462-5822.2012.01830.x 22735044

[B44] Lemire P. Houde M. Lecours M. P. Fittipaldi N. Segura M. (2012b). Role of capsular polysaccharide in Group B *Streptococccus* interactions with dendritic cells. Microbes Infect. 14, 1064–1076. 10.1016/j.micinf.2012.05.015 22683668

[B45] Lemire P. Roy D. Fittipaldi N. Okura M. Takamatsu D. Bergman E.. (2014). Implication of TLR- but not of NOD2-signaling pathways in dendritic cell activation by Group B *Streptococcus* serotypes III and V. PLoS ONE 9:e113940. 10.1371/journal.pone.0113940 25436906 PMC4250082

[B46] Lucas M. Schachterle W. Oberle K. Aichele P. Diefenbach A. (2007). Dendritic cells prime NK cells by trans-presenting IL-15. Immunity 26, 503–517. 10.1016/j.immuni.2007.03.006 17398124 PMC2084390

[B47] Maghazachi A. A. (2010). Role of chemokines in the biology of NK cells. Curr. Top. Microbiol. Immunol. 341, 37–58. 10.1007/82_2010_20 20369317

[B48] Maisey H. C. Doran K. S. Nizet V. (2008). Recent advances in understanding the molecular basis of Group B *Streptococcus* virulence. Expert Rev. Mol. Med. 10, e27. 10.1017/S1462399408000811 18803886 PMC2676346

[B49] Mancuso G. Cusumano V. Genovese F. Gambuzza M. Beninati C. Teti G. (1997). Role of IL-12 in experimental neonatal sepsis caused by Group B streptococci. Infect. Immun. 65, 3731–3735. 9284145 10.1128/iai.65.9.3731-3735.1997 PMC175532

[B50] Marcenaro E. Ferranti B. Falco M. Moretta L. Moretta A. (2008). Human NK cells directly recognize *Mycobacterium bovis* via TLR2 and acquire the ability to kill monocyte-derived dendritic cells. Int. Immunol. 20, 1155–1167. 10.1093/intimm/dxn073 18596023

[B51] McNeela E. A. Burke A. Neill D. R. Baxter C. Fernandes V. E. Ferreira D.. (2010). Pneumolysin activates the NLRP3 inflammasome and promotes proinflammatory cytokines independently of TLR4. PLoS Pathog. 6:e1001191. 10.1371/journal.ppat.1001191 21085613 PMC2978728

[B52] Mian M. F. Lauzon N. M. Andrews D. W. Lichty B. D. Ashkar A. A. (2010). FimH can directly activate human and murine NK cells via TLR4. Mol. Ther. 18, 1379–1388. 10.1038/mt.2010.75 20442710 PMC2911256

[B53] Mitchell A. J. Yau B. McQuillan J. A. Ball H. J. Too L. K. Abtin A.. (2012). Inflammasome-dependent IFN-gamma drives pathogenesis in *Streptococcus pneumoniae* meningitis. J. Immunol. 189, 4970–4980. 10.4049/jimmunol.1201687 23071286

[B54] Moretta L. Ferlazzo G. Bottino C. Vitale M. Pende D. Mingari M. C.. (2006). Effector and regulatory events during NK-dendritic cell interactions. Immunol. Rev. 214, 219–228. 10.1111/j.1600-065X.2006.00450.x 17100887

[B55] Mortier E. Woo T. Advincula R. Gozalo S. Ma A. (2008). IL-15Ralpha chaperones IL-15 to stable dendritic cell membrane complexes that activate NK cells via trans-presentation. J. Exp. Med. 205, 1213–1225. 10.1084/jem.20071913 18458113 PMC2373851

[B56] Newman K. C. Riley E. M. (2007). Whatever turns you on: accessory-cell-dependent activation of NK cells by pathogens. Nat. Rev. Immunol. 7, 279–291. 10.1038/nri2057 17380157

[B57] Nilsson N. Bremell T. Tarkowski A. Carlsten H. (1999). Protective role of NK1.1^+^ cells in experimental *Staphylococcus aureus* arthritis. Clin. Exp. Immunol. 117, 63–69. 10403917 10.1046/j.1365-2249.1999.00922.x PMC1905466

[B58] Okamura H. Kashiwamura S. Tsutsui H. Yoshimoto T. Nakanishi K. (1998). Regulation of IFN-gamma production by IL-12 and IL-18. Curr. Opin. Immunol. 10, 259–264. 9638361 10.1016/s0952-7915(98)80163-5

[B59] Pontiroli F. Dussurget O. Zanoni I. Urbano M. Beretta O. Granucci F.. (2012). The timing of IFN-beta production affects early innate responses to *Listeria monocytogenes* and determines the overall outcome of lethal infection. PLoS ONE 7:e43455. 10.1371/journal.pone.0043455 22912878 PMC3422257

[B60] Robertson M. J. (2002). Role of chemokines in the biology of NK cells. J. Leukoc. Biol. 71, 173–183. 11818437

[B61] Rosati E. Fettucciari K. Scaringi L. Cornacchione P. Sabatini R. Mezzasoma L.. (1998). Cytokine response to Group B *Streptococcus* infection in mice. Scand. J. Immunol. 47, 314–323. 10.1046/j.1365-3083.1998.00305.x 9600312

[B62] Roy D. Auger J. P. Segura M. Fittipaldi N. Takamatsu D. Okura M.. (2015). Role of the capsular polysaccharide as a virulence factor for *Streptococcus suis* serotype 14. Can. J. Vet. Res. 79, 141–146. 25852230 PMC4365706

[B63] Sanabria M. X. Vargas-Inchaustegui D. A. Xin L. Soong L. (2008). Role of NK cells in modulating dendritic cell responses to *Leishmania amazonensis* infection. Infect. Immun. 76, 5100–5109. 10.1128/IAI.00438-08 18794295 PMC2573361

[B64] Sancho D. Gomez M. Sanchez-Madrid F. (2005). CD69 is an immunoregulatory molecule induced following activation. Trends Immunol. 26, 136–140. 10.1016/j.it.2004.12.006 15745855

[B65] Schmidt K. N. Leung B. Kwong M. Zarember K. A. Satyal S. Navas T. A.. (2004). APC-independent activation of NK cells by the TLR3 agonist double-stranded RNA. J. Immunol. 172, 138–143. 10.4049/jimmunol.172.1.138 14688319

[B66] Segura M. (2012). Fisher scientific award lecture - the capsular polysaccharides of Group B *Streptococcus* and *Streptococcus suis* differently modulate bacterial interactions with dendritic cells. Can. J. Microbiol. 58, 249–260. 10.1139/w2012-003 22356626

[B67] Segura M. A. Cleroux P. Gottschalk M. (1998). *Streptococcus suis* and Group B *Streptococcus* differ in their interactions with murine macrophages. FEMS Immunol. Med. Microbiol. 21, 189–195. 10.1111/j.1574-695X.1998.tb01165.x 9718208

[B68] Slater J. D. Allen A. G. May J. P. Bolitho S. Lindsay H. Maskell D. J. (2003). Mutagenesis of *Streptococcus equi* and *Streptococcus suis* by transposon Tn917. Vet. Microbiol. 93, 197–206. 10.1016/S0378-1135(03)00030-0 12695044

[B69] Small C. L. McCormick S. Gill N. Kugathasan K. Santosuosso M. Donaldson N.. (2008). NK cells play a critical protective role in host defense against acute extracellular *Staphylococcus aureus* bacterial infection in the lung. J. Immunol. 180, 5558–5568. 10.4049/jimmunol.180.8.5558 18390740

[B70] Small C. L. Shaler C. R. McCormick S. Jeyanathan M. Damjanovic D. Brown E. G.. (2010). Influenza infection leads to increased susceptibility to subsequent bacterial superinfection by impairing NK cell responses in the lung. J. Immunol. 184, 2048–2056. 10.4049/jimmunol.0902772 20083661

[B71] Souza-Fonseca-Guimaraes F. Adib-Conquy M. Cavaillon J. M. (2012). NK cells in antibacterial innate immunity: angels or devils? Mol. Med. 18, 270–285. 10.2119/molmed.2011.00201 22105606 PMC3324953

[B72] Teti G. Mancuso G. Tomasello F. (1993). Cytokine appearance and effects of anti-TNF-alpha antibodies in a neonatal rat model of Group B streptococcal infection. Infect. Immun. 61, 227–235. 8418044 10.1128/iai.61.1.227-235.1993 PMC302709

[B73] Tettelin H. Masignani V. Cieslewicz M. J. Donati C. Medini D. Ward N. L.. (2005). Genome analysis of multiple pathogenic isolates of *Streptococcus agalactiae*: implications for the microbial “pan-genome”. Proc. Natl. Acad. Sci. U.S.A. 102, 13950–13955. 10.1073/pnas.0506758102 16172379 PMC1216834

[B74] Tsujimoto H. Uchida T. Efron P. A. Scumpia P. O. Verma A. Matsumoto T.. (2005). Flagellin enhances NK cell proliferation and activation directly and through dendritic cell-NK cell interactions. J. Leukoc. Biol. 78, 888–897. 10.1189/jlb.0105051 16033815

[B75] Van Calsteren M. R. Gagnon F. Calzas C. Goyette-Desjardins G. Okura M. Takamatsu D.. (2013). Structure determination of *Streptococcus suis* serotype 14 capsular polysaccharide. Biochem. Cell Biol. 91, 49–58. 10.1139/bcb-2012-0036 23527632

[B76] Van Calsteren M. R. Gagnon F. Lacouture S. Fittipaldi N. Gottschalk M. (2010). Structure determination of *Streptococcus suis* serotype 2 capsular polysaccharide. Biochem. Cell Biol. 88, 513–525. 10.1139/o09-170 20555393

[B77] Venet F. Davin F. Guignant C. Larue A. Cazalis M. A. Darbon R.. (2010). Early assessment of leukocyte alterations at diagnosis of septic shock. Shock 34, 358–363. 10.1097/SHK.0b013e3181dc0977 20220566

[B78] Wennekamp J. Henneke P. (2008). Induction and termination of inflammatory signaling in Group B streptococcal sepsis. Immunol. Rev. 225, 114–127. 10.1111/j.1600-065X.2008.00673.x 18837779 PMC5407011

[B79] Wesselkamper S. C. Eppert B. L. Motz G. T. Lau G. W. Hassett D. J. Borchers M. T. (2008). NKG2D is critical for NK cell activation in host defense against *Pseudomonas aeruginosa* respiratory infection. J. Immunol. 181, 5481–5489. 10.4049/jimmunol.181.8.5481 18832705 PMC2567053

[B80] Wilson C. B. (1986). Immunologic basis for increased susceptibility of the neonate to infection. J. Pediatr. 108, 1–12. 3511202 10.1016/s0022-3476(86)80761-2

[B81] Ye C. Zheng H. Zhang J. Jing H. Wang L. Xiong Y.. (2009). Clinical, experimental, and genomic differences between intermediately pathogenic, highly pathogenic, and epidemic *Streptococcus suis*. J. Infect. Dis. 199, 97–107. 10.1086/594370 19016627

[B82] Yea S. S. So L. Mallya S. Lee J. Rajasekaran K. Malarkannan S.. (2014). Effects of novel isoform-selective phosphoinositide 3-kinase inhibitors on NK cell function. PLoS ONE 9:e99486. 10.1371/journal.pone.0099486 24915189 PMC4051752

[B83] Yun C. H. Lundgren A. Azem J. Sjoling A. Holmgren J. Svennerholm A. M.. (2005). NK cells and *Helicobacter pylori* infection: bacterial antigens and IL-12 act synergistically to induce gamma interferon production. Infect. Immun. 73, 1482–1490. 10.1128/IAI.73.3.1482-1490.2005 15731046 PMC1064934

[B84] Zanoni I. Spreafico R. Bodio C. Di Gioia M. Cigni C. Broggi A.. (2013). IL-15 cis presentation is required for optimal NK cell activation in lipopolysaccharide-mediated inflammatory conditions. Cell Rep. 4, 1235–1249. 10.1016/j.celrep.2013.08.021 24055061

