# Supplementary material for: Natural Killer Cell Functions during the Innate Immune Response to Pathogenic Streptococci
Source: Front Microbiol. 2017 Jun 29;8:1196. doi: 10.3389/fmicb.2017.01196 (PMC5489694; doi:10.3389/fmicb.2017.01196)
Supplement: Supplementary file 1 [file Presentation1.pdf]

Supplementary Material

Natural Killer Cell Functions During the Innate Immune Response to Pathogenic Streptococci

Paul Lemire, Tristan Galbas, Jacques Thibodeau, and Mariela Segura*

*** Correspondence:** Mariela Segura: mariela.segura@umontreal.ca

Supplementary Figures

Figure S1. Purity levels of culture-expanded natural killer (NK) cells
Figure S2. Determination of optimal DC: NK ratio for co-cultures
Figure S3. Efficacy of *in vivo* depletion of NK cells
Figure S4. Bacteremia levels in GBS- or *S. suis*-infected mice after different starting doses of infection
Figure S5. Effect of *in vivo* NK1.1^{+} cell depletion on GBS or *S. suis* infection is dose-independent or –dependent, respectively
Figure S6. Effect of bacterial CPS on cytokine release by DCs in response to *S. suis*

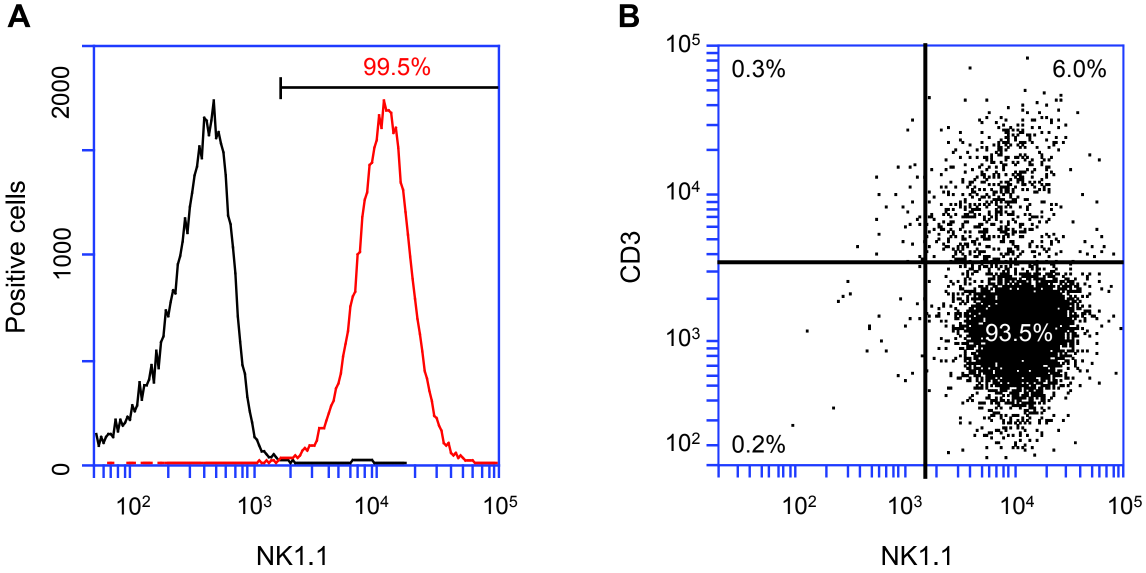

Supplementary Figure S1. **Purity levels of culture-expanded natural killer (NK) cells**. Untouched NK cells were purified from spleens of naive C57BL/6 mice by negative selection using magnetically activated cell sorting. Purified NK cells were expanded *in vitro* for 8 d at 37 °C with 5% CO_2 in complete RPMI 1640 medium supplemented with 500 ng/ml of recombinant mouse IL-2. **(A)** Histogram showing % of cells expressing NK1.1 surface marker as analyzed by FACS. **(B)** Dot plots showing % of $NK1.1^+$, $CD3^+$, double positive or double negative cell populations within the NK cell culture as analyzed by FACS.

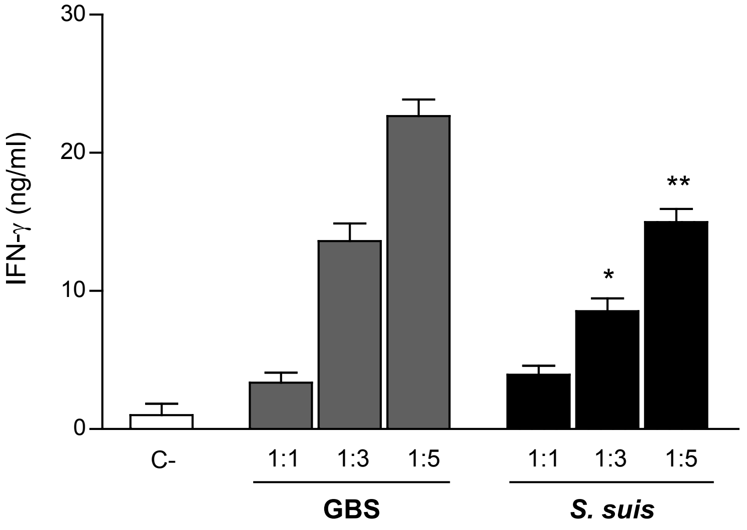

Supplementary Figure S2. **Determination of optimal DC: NK ratio for co-cultures**. IFN-γ levels were measured in DC and NK cell co-cultures established at ratios of 1:1, 1:3 or 1:5. Co-cultures were infected *in vitro* with either type III GBS strain COH-1 or type 2 *S. suis* strain P1/7 (2.5×10^5 CFU, initial DC-bacteria MOI:1). After bacterium-cell contact, antibiotics were added to prevent cell toxicity. Supernatants were collected at 14 h of incubation, and IFN-γ levels were measured by ELISA. Non-stimulated cells (medium alone) served as negative (C−) control. Data represent mean values (in ng/ml) ± SEM of three distinct experiments. *$P < 0.05$ or **$P < 0.01$, indicates statistically significant differences between type III GBS and type 2 *S. suis* strains for the same ratio.

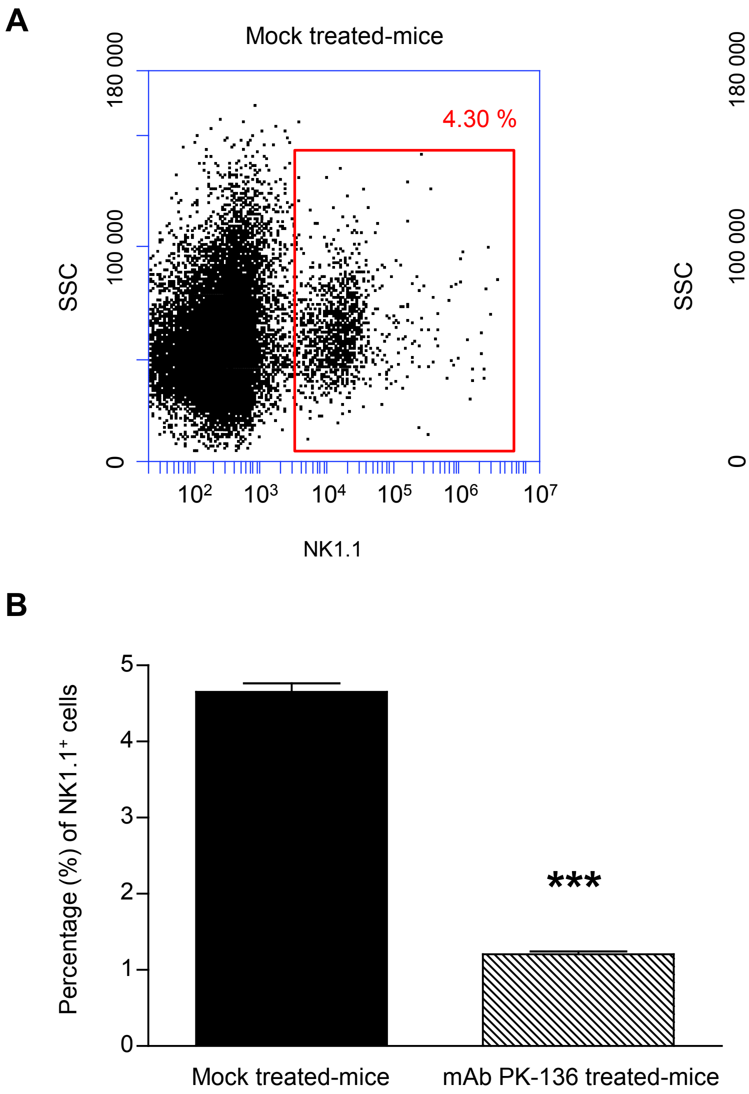

Supplementary Figure S3. Efficacy of *in vivo* depletion of NK cells. Mice were pre-treated intraperitoneally with isotype control in culture medium (mock-treated) or PK-136 monoclonal antibody (mAb) supernatant by 3 injections at day -4, -2 and -1 prior to infection. Twenty-four hours after the last injection (day 0), spleens were collected and cells were stained with PECy7-conjugated anti-NK1.1 mAb. (**A**) Representative dot plots showing % of NK1.1$^+$ cells in mock-treated or mAb PK-136 treated mice as analyzed by FACS. (**B**) Percentage of NK1.1$^+$ cells in mock-treated or mAb PK-136 treated mice. Data represent mean values ± SEM of 10 mice per group. ***$P < 0.0001$, indicates statistically significant differences between the two groups.

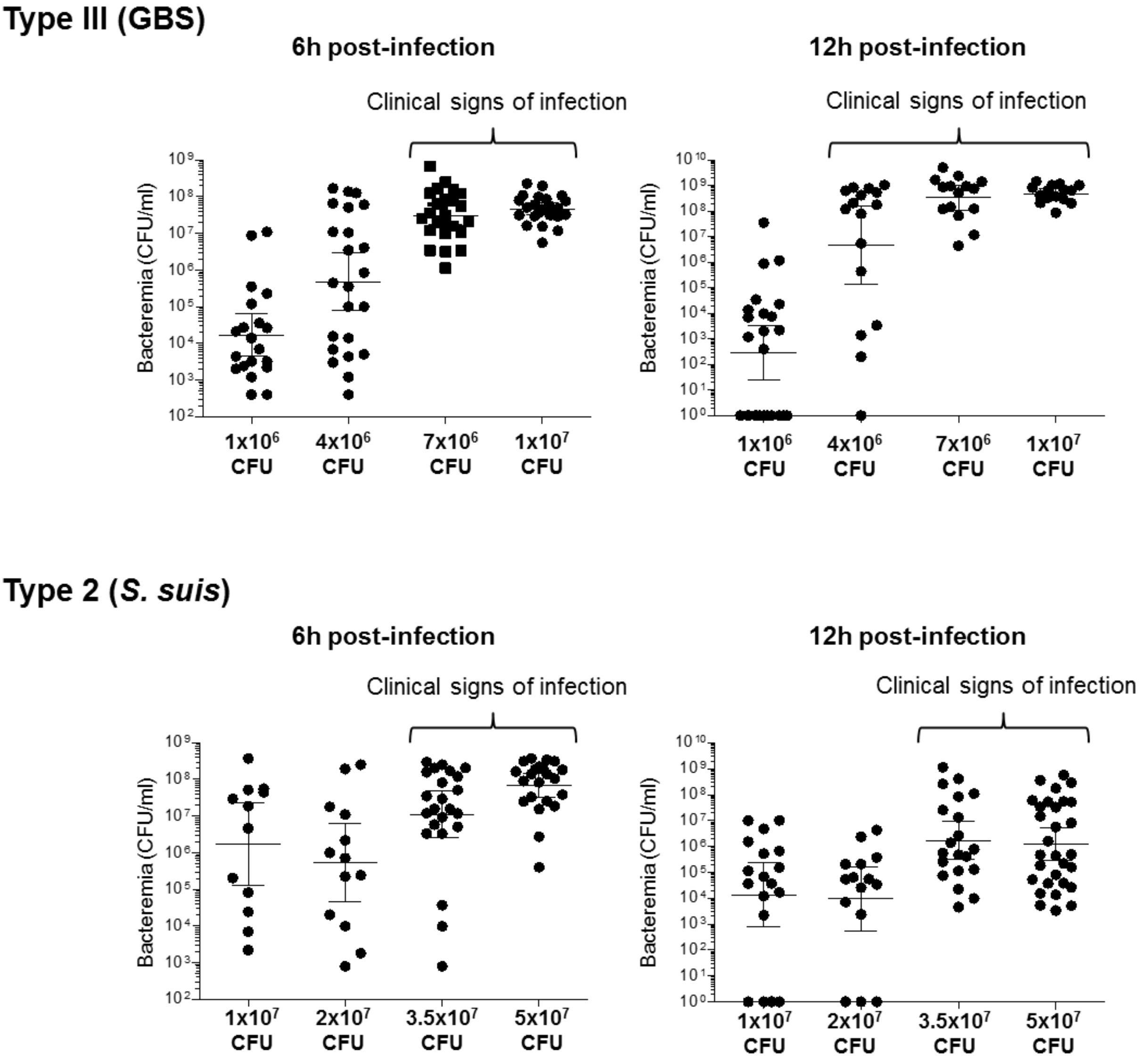

Supplementary Figure S4. Bacteremia levels in GBS- or *S. suis*-infected mice after different starting doses of infection. Mice were intraperitoneally infected with different doses of Group B *Streptococcus* (GBS) type III strain COH-1 and *Streptococcus suis* type 2 strain P1/7. Blood samples were collected from the tail at 6 h and 12 h post-infection, and plated onto THB agar plates. Colonies were counted and data expressed as CFU/ml of blood. Clinical signs of septic disease were also recorded, such as depression, swollen eyes, rough hair coat, and lethargy. N = 10-20 mice per group.

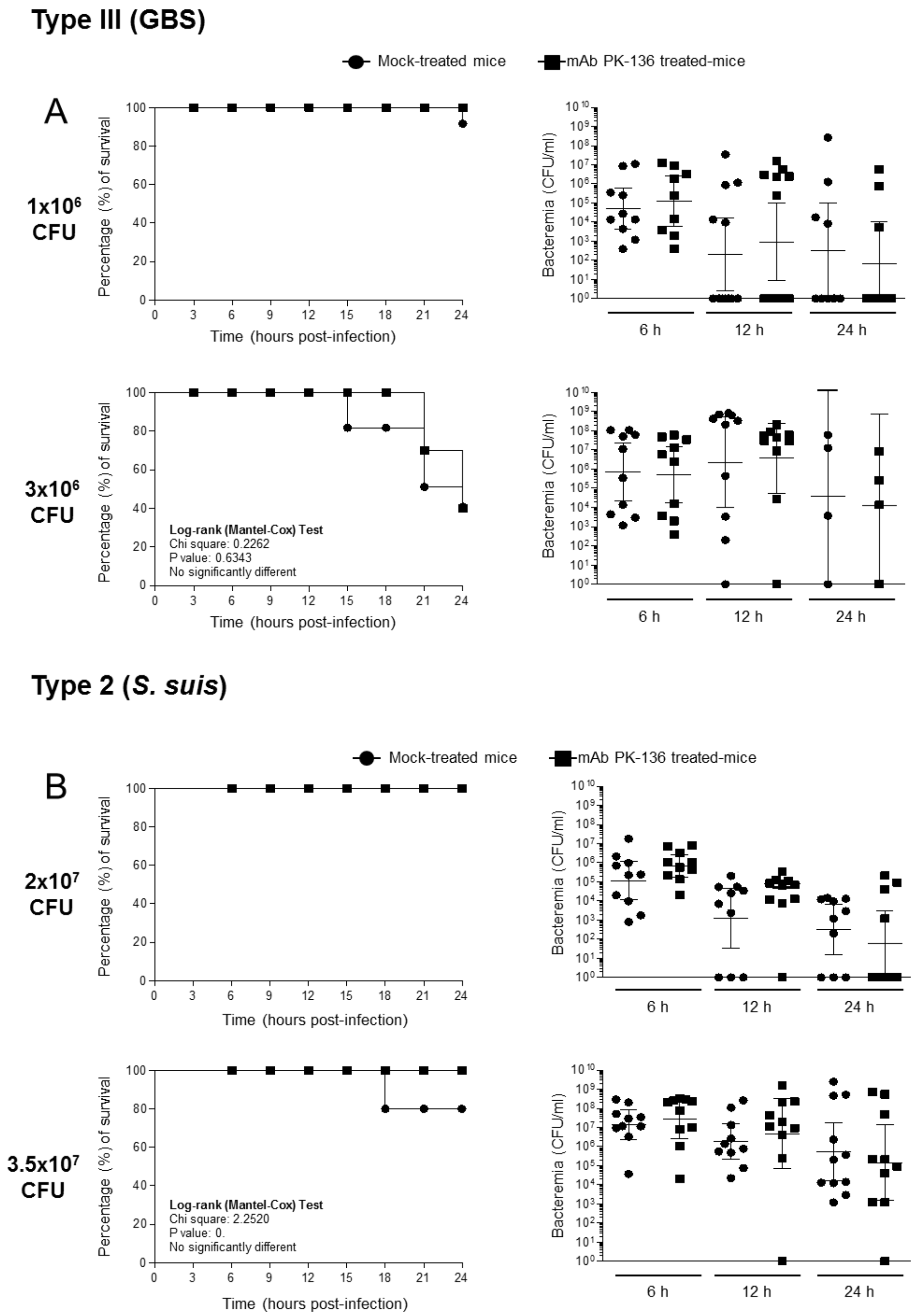

Supplementary Figure S5. Effect of *in vivo* NK1.1$^+$ cell depletion on GBS or *S. suis* infection is dose-independent or –dependent, respectively. Mice were either mock-treated or pre-treated with PK-136 monoclonal antibody (mAb) supernatant by 3 injections at days -4, -2 and -1 prior to infection. Twenty-four hours after the last injection (day 0), mice were intraperitoneally infected with different doses of type III GBS wild-type strain COH-1 or of type 2 *S. suis* wild-type strain P1/7 (n = 10). Survival and levels of bacteremia of infected mice were monitored during 24 h. Blood samples were collected from the tail at 6 h, 12 h, and 24 h post-infection, and plated onto THB agar plates. Colonies were counted and data expressed as CFU/ml of blood.

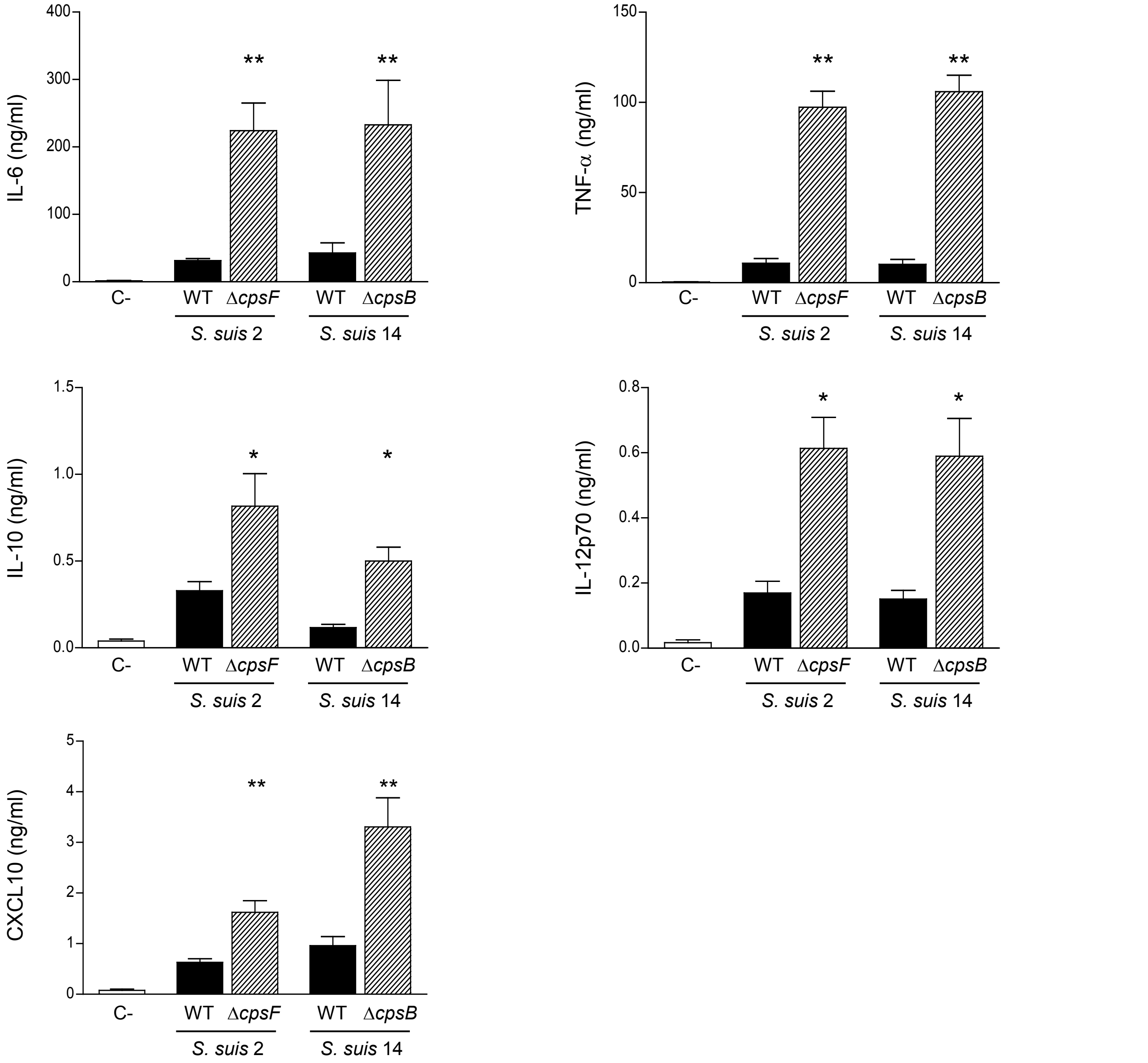

Supplementary Figure S6. **Effect of bacterial capsular polysaccharide (CPS) on cytokine release by DCs in response to *S. suis*.** DCs were stimulated with either type 2 or type 14 *S. suis* wild-type (WT) strains or their respective non-encapsulated (Δ*cps*) mutants (2.5 x 10^5 CFU, initial MOI:1). After bacterium-cell contact, antibiotics were added to prevent cell toxicity. Non-stimulated cells served as negative control (C-) for basal expression levels. Supernatants were harvested at 14 h of incubation and cytokine production quantified by ELISA. Data are expressed as mean ± SEM (in ng/ml) from eight independent experiments. *$P < 0.05$ or **$P < 0.01$, indicates statistically significant differences between WT strains and their respective non-encapsulated mutants.
